# Development of genome-wide polygenic risk scores for lipid traits and clinical applications for dyslipidemia, subclinical atherosclerosis, and diabetes cardiovascular complications among East Asians

**DOI:** 10.1186/s13073-021-00831-z

**Published:** 2021-02-19

**Authors:** Claudia H. T. Tam, Cadmon K. P. Lim, Andrea O. Y. Luk, Alex C. W. Ng, Heung-man Lee, Guozhi Jiang, Eric S. H. Lau, Baoqi Fan, Raymond Wan, Alice P. S. Kong, Wing-hung Tam, Risa Ozaki, Elaine Y. K. Chow, Ka-fai Lee, Shing-chung Siu, Grace Hui, Chiu-chi Tsang, Kam-piu Lau, Jenny Y. Y. Leung, Man-wo Tsang, Grace Kam, Ip-tim Lau, June K. Y. Li, Vincent T. F. Yeung, Emmy Lau, Stanley Lo, Samuel Fung, Yuk-lun Cheng, Chun-chung Chow, Miao Hu, Weichuan Yu, Stephen K. W. Tsui, Yu Huang, Huiyao Lan, Cheuk-chun Szeto, Nelson L. S. Tang, Maggie C. Y. Ng, Wing-yee So, Brian Tomlinson, Juliana C. N. Chan, Ronald C. W. Ma, The Hong Kong Diabetes Register TRS Study Group, The Hong Kong Diabetes Biobank Study Group

**Affiliations:** 1 Department of Medicine and Therapeutics, The Chinese University of Hong Kong, Hong Kong, China; 2 Hong Kong Institute of Diabetes and Obesity, The Chinese University of Hong Kong, Hong Kong, China; 3 CUHK-SJTU Joint Research Centre in Diabetes Genomics and Precision Medicine, Hong Kong, China; 4 Li Ka Shing Institute of Health Sciences, The Chinese University of Hong Kong, Hong Kong, China; 5 Department of Obstetrics and Gynaecology, The Chinese University of Hong Kong, Hong Kong, China; 6 Department of Medicine and Geriatrics, Kwong Wah Hospital, Yau Ma Tei, Hong Kong, China; 7 Diabetes Centre, Tung Wah Eastern Hospital, Causeway Bay, Hong Kong, China; 8 Diabetes and Education Centre, Alice Ho Miu Ling Nethersole Hospital, Tai Po, Hong Kong, China; 9 North District Hospital, Sheung Shui, Hong Kong, China; 10 Department of Medicine and Geriatrics, Ruttonjee Hospital, Wan Chai, Hong Kong, China; 11 Department of Medicine and Geriatrics, United Christian Hospital, Kwun Tong, Hong Kong, China; 12 Tseung Kwan O Hospital, Tseung Kwan O, Hong Kong, China; 13 Department of Medicine, Yan Chai Hospital, Tsuen Wan, Hong Kong, China; 14 Centre for Diabetes Education and Management, Our Lady of Maryknoll Hospital, Wong Tai Sin, Hong Kong, China; 15 Department of Medicine, Pamela Youde Nethersole Eastern Hospital, Chai Wan, Hong Kong, China; 16 Department of Medicine and Geriatrics, Princess Margaret Hospital, Lai Chi Kok, Hong Kong, China; 17 Department of Medicine, Alice Ho Miu Ling Nethersole Hospital, Tai Po, Hong Kong, China; 18 Department of Electronic and Computer Engineering, The Hong Kong University of Science and Technology, Clear Water Bay, Hong Kong, China; 19 School of Biomedical Sciences, The Chinese University of Hong Kong, Ma Liu Shui, Hong Kong, China; 20 Department of Chemical Pathology, The Chinese University of Hong Kong, Hong Kong, China; 21 Department of Medicine, Vanderbilt University Medical Center, Nashville, USA; 22 Faculty of Medicine, Macau University of Science and Technology, Taipa, Macau, China

**Keywords:** Polygenic risk scores, Lipid traits, Subclinical atherosclerosis, Diabetes cardiovascular complications, East Asians

## Abstract

**Background:**

The clinical utility of personal genomic information in identifying individuals at increased risks for dyslipidemia and cardiovascular diseases remains unclear.

**Methods:**

We used data from Biobank Japan (*n* = 70,657–128,305) and developed novel East Asian-specific genome-wide polygenic risk scores (PRSs) for four lipid traits. We validated (*n* = 4271) and subsequently tested associations of these scores with 3-year lipid changes in adolescents (*n* = 620), carotid intima-media thickness (cIMT) in adult women (*n* = 781), dyslipidemia (*n* = 7723), and coronary heart disease (CHD) (*n* = 2374 cases and 6246 controls) in type 2 diabetes (T2D) patients.

**Results:**

Our PRSs aggregating 84–549 genetic variants (0.251 < correlation coefficients (*r*) < 0.272) had comparably stronger association with lipid variations than the typical PRSs derived based on the genome-wide significant variants (0.089 < *r* < 0.240). Our PRSs were robustly associated with their corresponding lipid levels (7.5 × 10^− 103^ < *P* < 1.3 × 10^− 75^) and 3-year lipid changes (1.4 × 10^− 6^ < *P* < 0.0130) which started to emerge in childhood and adolescence. With the adjustments for principal components (PCs), sex, age, and body mass index, there was an elevation of 5.3% in TC (*β* ± SE = 0.052 ± 0.002), 11.7% in TG (*β* ± SE = 0.111 ± 0.006), 5.8% in HDL-C (*β* ± SE = 0.057 ± 0.003), and 8.4% in LDL-C (*β* ± SE = 0.081 ± 0.004) per one standard deviation increase in the corresponding PRS. However, their predictive power was attenuated in T2D patients (0.183 < *r* < 0.231). When we included each PRS (for TC, TG, and LDL-C) in addition to the clinical factors and PCs, the AUC for dyslipidemia was significantly increased by 0.032–0.057 in the general population (7.5 × 10^− 3^ < *P* < 0.0400) and 0.029–0.069 in T2D patients (2.1 × 10^− 10^ < *P* < 0.0428). Moreover, the quintile of TC-related PRS was moderately associated with cIMT in adult women (*β* ± SE = 0.011 ± 0.005, *P*_trend_ = 0.0182). Independent of conventional risk factors, the quintile of PRSs for TC [OR (95% CI) = 1.07 (1.03–1.11)], TG [OR (95% CI) = 1.05 (1.01–1.09)], and LDL-C [OR (95% CI) = 1.05 (1.01–1.09)] were significantly associated with increased risk of CHD in T2D patients (4.8 × 10^− 4^ < *P* < 0.0197). Further adjustment for baseline lipid drug use notably attenuated the CHD association.

**Conclusions:**

The PRSs derived and validated here highlight the potential for early genomic screening and personalized risk assessment for cardiovascular disease.

**Supplementary Information:**

The online version contains supplementary material available at 10.1186/s13073-021-00831-z.

## Background

Circulating lipids including levels of total cholesterol (TC), triglycerides (TG), high-density lipoprotein (HDL-C), and low-density lipoprotein (LDL-C) are among the most important, modifiable, and heritable risk factors for coronary heart disease (CHD). Previous studies have demonstrated a moderate-to-high heritability for variations in lipid levels, with estimates ranging from 20 to 60% [1]. Genome-wide association studies (GWASs) recently identified a number of common susceptibility variants for circulating lipids; however, the majority of these variants confer small risk individually and have limited predictive power for CHD risk [2].

It has been suggested that comprehensive genetic information could be used to quantify lifetime disease risk before the manifestation of clinical risk factors, contributing to risk stratification for clinical utility [3]. Although there were prior efforts to create polygenic risk scores (PRSs) for lipid traits, these traditionally comprised only of genetic variants reaching genome-wide significance, and only had limited success in improving CHD risk prediction [4, 5]. With the development of novel computational algorithms and the availability of large datasets, increasing number of PRSs for common diseases, which fully captured genome-wide variation, have been derived and validated [6, 7]. These approaches utilized full results from previous genome-wide association studies and an external reference panel to construct the PRSs mainly based on two strategies: (1) liberalization of the significance thresholds for variant inclusion while accounting for linkage disequilibrium (LD) patterns in a population; and (2) assignment of new weightings to variants using the Bayesian method that infers the posterior mean effect for each variant by assuming a prior effect from GWAS summary statistics, the information of genomic correlation, and a pre-specified proportion of causal variants. For example, Khera et al. recently constructed six genome-wide PRSs, incorporating information from 5218 to 6,917,436 common genetic variants, to predict the risks of developing CHD, atrial fibrillation, type 2 diabetes (T2D), inflammatory bowel disease, breast cancer, and severe obesity in participants of mostly European ancestry [7, 8].

To further investigate the potential use of genetic information in identifying and screening individuals at increased risks for dyslipidemia and diabetes cardiovascular complications, we applied the recently developed computational methods to optimize PRSs for four lipid traits in multiple cohorts of East Asians at various stages of the life-course, and subsequently tested their performance in the general population and patients with T2D. Moreover, we evaluated the effect of the best-performing PRSs on 3-year lipid changes in adolescents. Finally, we examined the potential clinical implication of these PRSs in subclinical atherosclerosis in adult women and coronary heart disease in T2D patients.

## Methods

### Study subjects

**Fig. 1:**
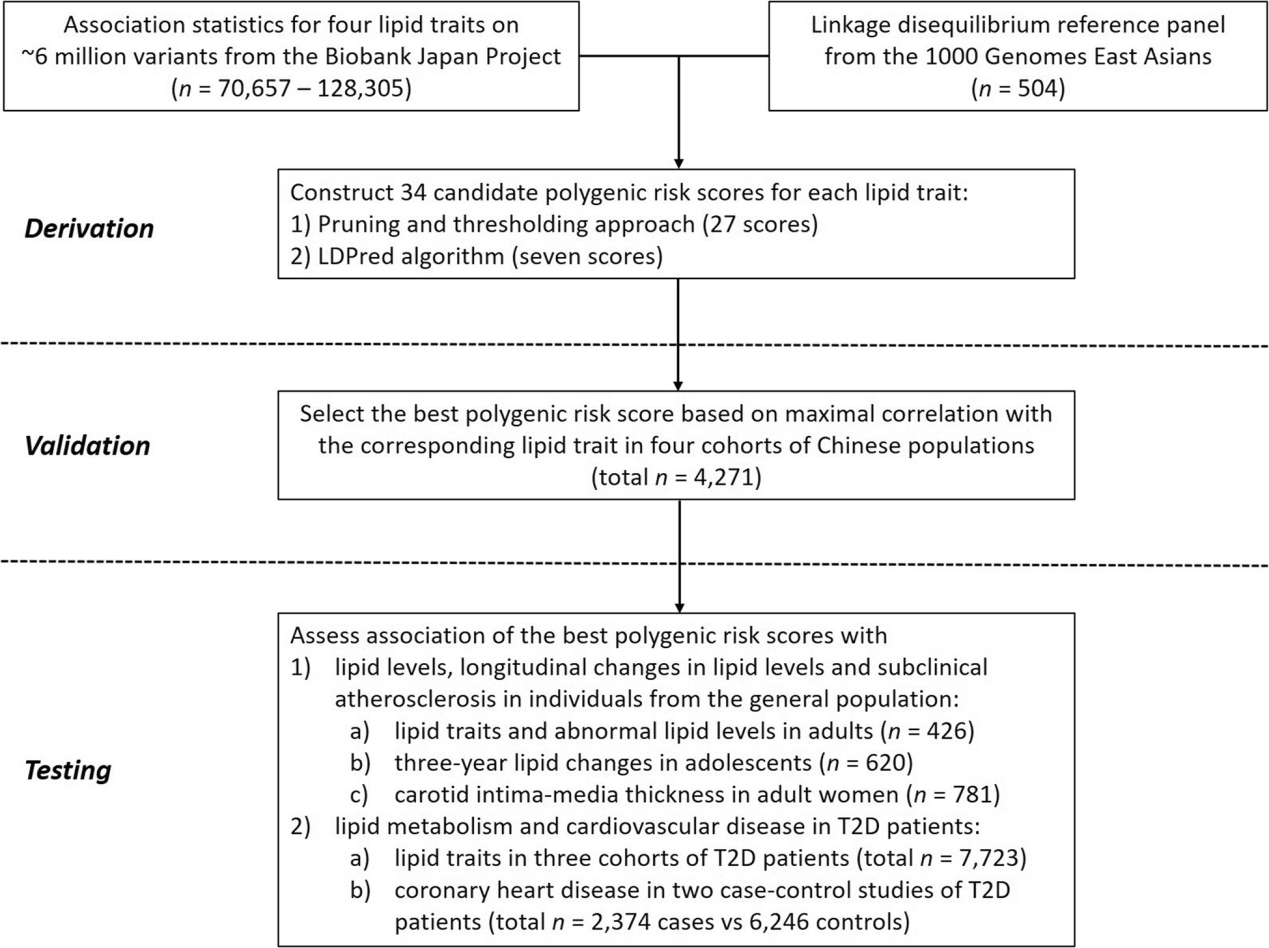
Study design and workflow. A polygenic risk score (PRS) for each lipid trait was derived by (1) association statistics from the Biobank Japanese Project and (2) linkage disequilibrium (LD) between genetic variants from a reference panel of 504 East Asians in 1000 Genomes Project. A total of 34 candidate PRSs were developed using two strategies: (1) the “pruning and thresholding” approach, which involves pruning the genetic variants based on the pairwise threshold of LD *r*^*2*^ (0.2, 0.4, and 0.6), and subsequently applying a *p* value threshold (1, 0.5, 0.1, 0.05, 0.01, 1 × 10^−3^, 1 × 10^−4^, 1 × 10^−5^, and 5 × 10^−8^) to the association statistics. And (2) the LDPred computational algorithm, a Bayesian method that estimates the posterior mean causal effect for each variant by assuming a prior effect size from summary statistics and LD information from an external reference panel. Multiple LDpred scores were calculated by varying the tuning parameter *ρ* (1, 0.3, 0.1, 0.03, 0.01, 3 × 10^− 3^, and 1 × 10^− 3^) which are the fractions of markers with non-zero effects. The optimal PRS for each lipid trait was chosen based on maximal correlation with the corresponding lipid trait in a total of 4271 individuals in the validation datasets, and then tested for the associations with lipid metabolism, changes in lipid levels, and cardiovascular risk in multiple independent cohorts

The design of this study is shown in Fig. 1. Participants included in the validation and testing datasets for assessing the predictive ability of PRSs and in the analyses for the cardiovascular outcome were of southern Han Chinese ancestry residing in Hong Kong.

Data used for the development of PRSs for four lipid traits came from the BioBank Japan (BBJ) Project [9], which is one of the largest non-European single-descent biobanks with detailed phenotypes. It comprised 128,305 Japanese individuals in the TC analysis, 105,597 individuals in the TG analysis, 70,657 individuals in the HDL-C analysis, and 72,866 individuals in the LDL-C analysis. Details of the study design of the BBJ Project have been previously described [10]. Briefly, the BBJ Project is a multi-institutional hospital-based registry that collected DNA, serum, and clinical information of approximately 200,000 patients from 66 hospitals affiliated with 12 medical institutes between fiscal years 2003 and 2007. All study participants had been diagnosed with one or more of the 47 target diseases (including lung cancer, esophageal cancer, gastric cancer, colorectal cancer, liver cancer, pancreatic cancer, gallbladder / cholangiocarcinoma, prostate cancer, breast cancer, uterine cervical cancer, uterine corpus cancer, ovarian cancer, hematological cancer, cerebral infarction, cerebral aneurysm, epilepsy, bronchial asthma, pulmonary tuberculosis, chronic obstructive pulmonary disease, interstitial lung disease / pulmonary fibrosis, myocardial infarction, unstable angina, stable angina, arrhythmia, heart failure, peripheral arterial diseases, chronic hepatitis B, chronic hepatitis C, liver cirrhosis, nephrotic syndrome, urolithiasis, osteoporosis, diabetes mellitus, dyslipidemia, graves’ disease, rheumatoid arthritis, hay fever, drug eruption, atopic dermatitis, keloid, uterine fibroid, endometriosis, febrile seizure, glaucoma, cataract, periodontitis, and amyotrophic lateral sclerosis) by physicians at the cooperating hospitals as described in the previous reports [10].

Details of the study design, ascertainment, inclusion criteria, and phenotyping procedures of the participants involved in the validation and testing stages are described in “*Cohort Descriptions*” (See Additional File 1: Supplementary Methods). Individuals who were receiving lipid-lowering medication at the time of examination were excluded from the data used to assess the predictive ability of PRSs for lipid traits. The validation dataset consists of 4271 individuals at different stages of the life-course from four cohorts of Chinese ancestry: (1) 909 children enrolled in the follow-up visit of the Hyperglycemia and Adverse Pregnancy Outcome (HAPO) study at the Hong Kong center [11]; (2) 1973 adolescents recruited from a community-based school survey for risk factor assessment [12]; (3) 441 healthy adults enlisted from hospital staff, a territory-wide health awareness, and promotion program selected by stratified random sampling with computer-generated codes in accordance to the distribution of occupational groups, and the community-based pharmacogenetics studies in hypertension and dyslipidemia [13, 14]; and (4) 948 adult women attended the HAPO follow-up study [11]. The best PRSs for lipid traits were further evaluated in four independent testing datasets, comprising 426 adults recruited from hospital staff, and a territory-wide health awareness and promotion program, as well as a total of 7723 individuals drawn from three prospective cohorts of Chinese patients with T2D: (1) 4917 patients from the Hong Kong Diabetes Register (HKDR), which was established as a quality improvement program at the Prince of Wales Hospital at the Chinese University of Hong Kong since 1995 [15]; (2) 1941 patients; and (3) 865 patients enrolled in the Hong Kong Diabetes Biobank (HKDB) phase 1 and phase 2 studies, respectively [16], which aims to establish a territory-wide registry and biobank of individuals with diabetes for large-scale genetic replication studies, biomarker discovery, and epidemiology research.

Analyses for the associations between PRSs and 3-year changes in lipid traits were performed in a subset of 620 adolescents who attended both the baseline and the follow-up assessment (baseline 2003–2004, follow-up 2006). In the analysis for subclinical atherosclerosis, a total of 781 adult women with carotid intima-media thickness (cIMT; a marker of subclinical atherosclerosis) measurement were drawn from two prospective cohorts primarily designed to assess the impact of gestational hyperglycemia on the pregnancy outcomes in women and offspring (*n* = 654 in the adult women of cohort 1 [11] and *n* = 127 in the adult women of cohort 2 [17]). We further evaluated the influence of PRSs for lipid traits on the risk of CHD using data generated from two prospective studies, the HKDR Study and the HKDB Study. A total of 2374 cases with T2D and CHD, and 6246 T2D patients without CHD events were examined.

### Outcome variables

In the BBJ Project, the measurements of TC, TG, and HDL-C were retrieved from medical records. LDL-C were either retrieved from medical records or derived from the Friedewald’s formula as TC − HDL-C − (TG / 2.2) when LDL-C is not available and TG < 4.5 mmol/l [9, 10].

All participants included in the validation and testing stages were examined in the morning after an overnight fast. Fasting blood samples were collected for the measurements of lipid profiles (TC, TG, HDL-C, and calculated LDL-C). TC (enzymatic method), TG (enzymatic method without glycerol blanking), and HDL-C (direct method using PEG-modified enzymes and dextran sulfate) were measured on a Roche Modular Analytics system (Roche Diagnostics GmbH, Mannheim, Germany) using standard reagent kits supplied by the manufacturer of the analyzer. LDL-C was calculated by using Friedewald’s formula for TG < 4.5 mmol/1 [18]. Among the adolescents who attended both the baseline and the follow-up study, we used the longitudinal data on lipid levels to calculate the 3-year changes in four lipid traits as (lipid_follow-up_ − lipid_baseline_) / lipid_baseline_.

Dyslipidemia/abnormal lipid levels were defined according to the thresholds used in clinical practice guidelines [19]: (1) TC ≥ 5.1 mmol/l; TG ≥ 1.1 mmol/l; and LDL-C ≥ 3.4 mmol/l in children; (2) TC ≥ 5.1 mmol/l; TG ≥ 1.4 mmol/l; and LDL-C ≥ 3.4 mmol/l in adolescents; (3) TC ≥ 5.2 mmol/l; TG ≥ 1.7 or ≥ 1.97 mmol/l; and LDL-C ≥ 1.8 or ≥ 2.6 mmol/l in adults or patients with T2D.

In the two cohorts of adult women, cIMT was measured with a L12–5-MHz linear transducer using methodology described in our previous study [20]. Three cIMT measurements were made in the plaque-free section of both right and left common carotid arteries, along the thickest point on the far wall and within approximately 1.5 cm proximal to the flow divider. The mean cIMT was calculated by averaging six measurements from both sides. The intra-class correlation coefficients for inter- and intraoperator reliability for cIMT measurement were 0.98 (95% CI 0.93–1.0) and 0.98 (0.91–0.99), respectively.

Coronary heart disease (CHD) outcome was defined based on the discharge principal diagnoses of hospital admissions and mortality until June 2017. We retrieved the data of hospital admissions from the Hong Kong Hospital Authority Central Computer System, which records the admissions to all public hospitals as well as deaths and causes of death. Hospital discharge principal diagnoses coded by the International Classification of Diseases, Ninth Revision (ICD-9) were used to identify the outcome event. The CHD ascertainment was based on a composite of (1) acute myocardial infarction (code 410); or (2) nonfatal ischemic heart disease (codes 411 to 414); or (3) death due to CHD (not including death due to heart failure), which occurred either at baseline or during follow-up. Among the T2D patients from the HKDR and HKDB studies, we have examined a total of 2374 CHD cases and 6246 controls who had duration of T2D more than 10 years and were free from cardiovascular diseases including CHD, stroke, and peripheral vascular disease.

### Genotyping, quality control, and imputation

Individuals in the BBJ project underwent genotyping with either the Illumina HumanOmniExpressExome BeadChip or a combination of the Illumina HumanOmniExpress and HumanExome BeadChips. Exclusion criteria for samples and quality control (QC) criteria for single nucleotide polymorphisms (SNPs) have been previously reported [9]. Genotype data were imputed to the 1000 Genomes Project Phase 1 v3 East Asian reference panel using minimac [21]. Imputed SNPs with an imputation quality *r*^*2*^ < 0.7 were excluded from the subsequent association analysis.

DNA samples included in the validation and testing stages were genotyped using one of four arrays: (1) Illumina Omni2.5 + Exome Array, (2) Illumina HumanOmni ZhongHua-8 BeadChip, (3) Infinium® Asian Screening Array, and (4) Infinium® Global Screening Array. We have applied the same standard QC procedures on each genome-wide SNP array data. The per-individual QC of genotype data consists of four steps: (1) sex checking based on the genotype call from chromosome X; (2) detection of low-quality samples based on call rate and heterozygosity rate; (3) detection of possible familial relationship or duplicated individuals using estimates of identity-by-descent (IBD); (4) detection of population stratification by performing principal component (PC) analysis (See Additional file 1: Fig. S1). Only biallelic autosomal SNPs were included in the per-marker QC. SNPs were excluded from further analysis if (1) Hardy–Weinberg equilibrium (HWE) *p* < 1 × 10^− 4^ and (2) minor allele frequency (MAF) < 1%; or 3) call rate < 95%. In particular, SNPs with MAF ≥ 1% but ≤ 5% are excluded if their call rate is < 99%.

Within each individual cohort, we imputed the genotype data to the 1000 Genomes Project phase III reference panel (October 2014) using the Michigan Imputation Server [22]. SNPs with MAF < 1%, imputation quality score *r*^*2*^ < 0.5, or ambiguous strands (A/T or C/G) were removed. Finally, ~ 4.5 million SNPs overlapped among all derivation and validation datasets were included in the score derivation. In the testing datasets, all SNPs used in the calculation of PRSs had an imputation quality score *r*^*2*^ > 0.3.

### Construction of polygenic score

In general, the form of a PRS is *β*_*1*_*x*_*1*_ + *β*_*2*_*x*_*2*_ + … + *β*_*k*_*x*_*k*_ + … + *β*_*n*_*x*_*n*_ where *β*_*k*_ is the per-allele effect size for lipid level associated with SNP *k*, *x*_*k*_ is an indication function of the effect allele (e.g., the number of effect alleles) at SNP *k*, and *n* is the total number of SNPs involved in the candidate PRS. To derive the PRS for each lipid trait, we used (1) publicly available association statistics (including the effect allele, the estimated *β*-coefficient for the effect allele, and the *p* value of each genetic variant) from a recent genome-wide association study (GWAS) in the Japanese population contributed by the BBJ Project [9] and (2) LD between genetic variants from a reference panel of 504 East Asians contributed by the 1000 Genomes Project [23]. For each lipid traits, a total of 34 candidate PRSs were built using two different strategies.

The first 27 PRSs were constructed by the “pruning and thresholding” approach, which was implemented using the “clumping” procedure in PLINK v1.90 [24]. This is a greedy algorithm, iteratively choosing a set of SNPs to form clumps around the index SNPs [i.e., these SNPs are significant at a provided *p* value threshold (1, 0.5, 0.1, 0.05, 0.01, 1 × 10^− 3^, 1 × 10^− 4^, 1 × 10^− 5^, and 5 × 10^− 8^) in the BBJ GWAS]. Each clump is composed of SNPs which are within 250 kb from the index SNP and are also in LD with the index SNP based on the pairwise threshold of *r*^*2*^ (0.2, 0.4, and 0.6) [7]. Given a threshold of *p* value and *r*^*2*^, a candidate PRS was computed based on the resultant index SNPs of each clump and the corresponding estimated *β*-coefficient for its effect allele as weights using the “score” procedure in PLINK v2.0 [24].

Seven additional PRSs were developed by the LDPred computational algorithm, a Bayesian method that estimates the posterior mean causal effect for each variant by assuming a prior effect size from summary statistics (e.g., association statistics from the BBJ GWAS) and LD information from an external reference panel (e.g., LD reference panel from the 1000 Genomes East Asians) [25]. Multiple LDpred scores were calculated by varying the tuning parameter *ρ* (1, 0.3, 0.1, 0.03, 0.01, 3 × 10^− 3^, and 1 × 10^− 3^), which are the fractions of markers with non-zero effects. It is recommended to include the 1.2 M HapMap3 SNPs for this analysis. Thus the number of variants was down sized to 902,892, using only the variants included within the HapMap3 data (https://www.broadinstitute.org/medical-and-population-genetics/hapmap-3) and overlapped among all derivation and validation datasets.

Optimal PRS for each lipid trait was chosen based on maximal pooled Pearson correlation with the corresponding measured lipid trait in a total of 4271 individuals in validation datasets. The best-performing PRS for each lipid trait was transformed to a *z*-score and then further classified into five categories using the quintile thresholds defined in the largest cohort (e.g., the HKDR cohort) in this study. These scores and their quintiles were then tested for the associations with (1) corresponding lipid level in adults from general population and T2D patients (testing datasets), (2) 3-year changes in corresponding lipid level in adolescents, (3) cIMT in adult women, and (4) the risk of CHD in T2D patients.

Additional PRSs for four lipid traits comprised of (1) only the lead variants and (2) both the lead and independent variants previously reaching genome-wide significance in European populations were generated to compare the predictive power with the best-performing PRSs derived in the current study [2, 26–32]. Only 85 TC-related, 87 TG-related, 102 HDL-C-related, and 70 LDL-C-related lead variants were available in our datasets. When both the lead variants and the independent variants were considered, the numbers of variants associated with TC, TG, HDL-C, and LDL-C were increased to 229, 259, 328, and 201, respectively.

### Statistical analysis

All analyses were performed using PLINK v1.9 (https://www.cog-genomics.org/plink/1.9/, 31 December, 2019) and v2.0 (https://www.cog-genomics.org/plink/2.0/, 31 December, 2019) [24], LDpred v1.0.6 software package [25], IBM SPSS Statistics 25, and R 3.4.4 (http://www.r-project.org/, 31 December, 2019) unless specified otherwise. A 2-tailed *p* value < 0.05 was considered statistically significant. Data are presented as percentages (*n*), mean ± SD, or geometric mean (95% CI). Comparison between groups was performed by chi-squared test, unpaired Student’s T-test, or Mann-Whitney test, as appropriate.

Within each cohort, associations between PRSs and lipid traits were assessed by Pearson and Spearman correlations, and linear regression with the adjustment of PCs, sex, age, and body mass index (BMI). A pooled correlation across individual cohorts was calculated using the Fisher Z transformation approach [33]. Results for either linear or logistic regression from individual cohorts were combined by inverse-variance weighted meta-analysis using a fixed effects model. The proportion of variance for a lipid trait explained by the corresponding optimal PRS was computed as the *R*^*2*^ obtained from a full model including both PRS and covariates (PCs, sex, age, and BMI) minus the *R*^*2*^ obtained from a model including covariates alone. The best-performing PRSs were tested for associations with the 3-year changes in lipid levels in adolescents and cIMT in adult women using linear regression adjusted for covariates. In the analysis for 3-year changes, we adjusted for PCs, sex, and age at follow-up, BMI at baseline and follow-up, and the corresponding lipid trait at baseline. In the analysis for cIMT, we adjusted for (1) PCs and age and (2) PCs, age, BMI, and systolic blood pressure (SBP). To examine the association between the best-performing PRSs for lipid traits and the risk of CHD in T2D patients, we further conducted a logistic regression analysis adjusted for covariates as follows: model 1 included PCs, sex, age, and duration of diabetes; model 2 included the covariates in model 1 and BMI; model 3 included the covariates in model 2 and smoking status; model 4 included the covariates in model 3, HbA_1c_, and SBP; model 5 included the covariates in model 4, estimated glomerular filtration rate (eGFR), and log-transformed albumin-creatinine ratio (ACR); model 6 included the covariates in model 5 and the use of lipid-lowering drugs.

To evaluate the discriminative power of our best PRSs to identify those with clinically defined dyslipidemia, we calculated the area under the receiver operating characteristic (ROC) curve, denoted as the area under curve (AUC) based on the predicted risks for each individual obtained from the logistic regression analysis. The AUC can vary from 0.5 (no discrimination) to 1 (prefect discrimination). Moreover, we presumed that associations for lipid measurements may be confounded by some clinical risk factors (e.g., sex, age, and BMI). Therefore, we explored whether our PRSs predict the risk of dyslipidemia independently of the clinical risk factors. Three different models were considered: model 1—sex, age, BMI, and PCs; model 2—PRS only; and model 3—sex, age, BMI, PCs, and PRS. The contribution of PRS to AUC on top of sex, age, BMI, and PCs was computed as the AUC obtained from model 3 minus the AUC obtained from model 1. We compared two correlated AUCs using the DeLong method [34].

We further calculated the positive predictive value (PPV), negative predictive value (NPV), sensitivity and specificity of high PRS (top 20% vs. the remaining 80% of the PRS distribution) to assess their precision for diagnosing dyslipidemia. PPV is the proportion of individuals who actually have the disease among all those who have a positive prediction (i.e., true positive/[true positive + false positive]). Negative predictive value is the proportion of individuals who actually do not have that disease among all those who have a negative prediction (i.e., true negative/[true negative + false negative]). Sensitivity is the proportion of individuals who have a positive prediction among all those who actually have the disease (i.e., true positive/[true positive + false negative]). Specificity is the proportion of individuals who have a negative prediction among all those who actually do not have that disease (i.e., true negative/[true negative + false positive]). In this analysis, a positive prediction is the prediction that an individual has a high PRS (top 20% of the PRS distribution), while a negative prediction is the prediction that an individual has a low PRS (remaining 80% of the PRS distribution).

## Results

### Derivation, validation, and testing of PRSs for four lipid traits

The clinical characteristic of the individuals who were involved in assessing the predictive performance of PRSs for lipid traits (*n* = 4271 and 8149 in validation and testing datasets, respectively) is depicted in Additional file 2: Table S1. By using the association statistics from the BBJ Project and the LD reference panel from 1000 Genomes East Asians, we utilized two different methods to build 34 candidate PRSs for each lipid trait: (1) the first 27 PRSs were derived based on a pruning and thresholding approach, and (2) 7 additional PRSs were developed using the recently proposed LDPred computational algorithm (See Fig. 1). We validated these scores in 4271 individuals from four cohorts at different stages of the life-course (childhood, adolescence, and adulthood) and chose the best-performing PRSs for each lipid trait by selecting the PRS which had the maximum pooled Pearson correlation with the corresponding measured lipid trait (See Additional File 1: Fig. S2, and Additional File 2: Tables S2–5). Proportion of phenotypic variance in lipid levels explained by each candidate PRSs are shown in Additional File 1: Fig. S3.

Here we report results for PRSs giving the highest prediction accuracy (See Table 1 and Additional File 1: Fig. S4). The four optimal PRSs for TC, TG, HDL-C, and LDL-C were derived by the pruning and thresholding approach, comprising of 229, 142, 549, and 84 SNPs, respectively. All the SNPs included in TG- and LDL-C-related PRSs achieved genome-wide significance in the BBJ study (*P* = 5.0 × 10^− 8^), whereas only 95 (58.5%) and 231 (42.1%) SNPs were previously reported as genome-wide significant in the TC- and HDL-C-related PRSs, respectively. These PRSs were robustly associated with their corresponding measured lipid levels, with pooled correlation coefficients ranging from 0.256 for TG to 0.304 for TC. The meta-analysis results demonstrated an increase of 5.3% in TC (*P* = 7.5 × 10^− 103^), 11.7% in TG (*P* = 1.3 × 10^− 75^), 5.8% in HDL-C (*P* = 9.3 × 10^− 83^), and 8.4% in LDL-C (*P* = 2.4 × 10^− 93^) per one standard deviation (1-SD) increase in the corresponding PRS, after adjusting for PCs sex, age, and BMI. The proportion of phenotypic variance in lipid levels explained by the corresponding PRSs ranged from 6.3 to 10.9% for TC, 5.6 to 8.6% for TG, 6.4 to 9.4% for HDL-C, and 6.3 to 10.9% for LDL-C in validation datasets.

**Table 1:** Best-performing polygenic risk scores and testing for associations with measured lipid traits

Polygenic risk score	Lipid trait	No. of SNPs	Derivation strategy and tuning parameter	Cohort	***n***	Correlation with lipid trait		Linear regression				Proportion of variance
						Pearson ***r***	Spearman ***r***	***β***	SE	***P***	***P***_***Q***_	explained by polygenic risk score
PRS_TC_	TC	229	Pruning and thresholding	Children	909	0.299	0.295	0.050	0.005	2.2 × 10^−21^	–	9.49%
			(*r*^2^ = 0.2 and *p* = 10^− 5^)	Adolescents	1973	0.327	0.322	0.056	0.004	8.9 × 10^− 53^	–	10.93%
				Healthy adults	441	0.334	0.320	0.057	0.008	2.2 × 10^−13^	–	10.07%
				Adult women	948	0.243	0.259	0.042	0.005	3.2 × 10^−16^	–	6.26%
				***Combined analysis in validation dataset***	**4271**	**0.304**	**0.302**	**0.052**	**0.002**	**7.5 × 10**^**−103**^	**0.1197**	–
				**Adults**	**426**	**0.251**	**0.263**	**0.043**	**0.008**	**2.9 × 10**^**−7**^	**–**	5**.**06%
				T2D patients in HKDR study	4917	0.186	0.199	0.042	0.003	3.7 × 10^−45^	–	3.93%
				T2D patients in HKDB phase 1 study	1941	0.187	0.186	0.036	0.004	2.3 × 10^−16^	–	3.32%
				T2D patients in HKDB phase 2 study	865	0.173	0.176	0.034	0.006	1.8 × 10^−7^	–	2.98%
				***Combined analysis in T2D patients***	**7723**	**0.185**	**0.193**	**0.039**	**0.002**	**1.5 × 10**^**−66**^	**0.3721**	–
PRS_TG_	TG	142	Pruning and thresholding	Children	909	0.243	0.245	0.106	0.012	3.7 × 10^−17^	–	7.19%
			(*r*^2^ = 0.2 and *p* = 5 × 10^−8^)	Adolescents	1973	0.244	0.227	0.097	0.008	3.4 × 10^−30^	–	5.99%
				Healthy adults	441	0.257	0.245	0.139	0.022	9.5 × 10^−10^	–	5.59%
				Adult women	948	0.294	0.256	0.146	0.015	9.9 × 10^−23^	–	8.59%
				***Combined analysis in validation dataset***	**4271**	**0.256**	**0.239**	**0.111**	**0.006**	**1.3 × 10**^**−75**^	**0.0150**	–
				**Adults**	**426**	**0.251**	**0.230**	**0.133**	**0.023**	**1.7 × 10**^**−8**^	–	5**.**14%
				T2D patients in HKDR study	4917	0.200	0.251	0.165	0.008	1.3 × 10^−87^	–	7.31%
				T2D patients in HKDB phase 1 study	1941	0.209	0.191	0.133	0.013	1.6 × 10^−25^	–	5.00%
				T2D patients in HKDB phase 2 study	865	0.231	0.197	0.140	0.019	2.8 × 10^−13^	–	5.58%
				***Combined analysis in T2D patients***	**7723**	**0.206**	**0.230**	**0.154**	**0.006**	**2.3 × 10**^**−126**^	**0.0721**	–
PRS_HDL_	HDL-C	549	Pruning and thresholding	Children	909	0.263	0.250	0.052	0.007	4.0 × 10^−15^	–	6.37%
			(*r*^2^ = 0.2 and *p* = 10^−4^)	Adolescents	1973	0.281	0.258	0.054	0.004	4.0 × 10^− 38^	–	7.27%
				Healthy adults	441	0.347	0.313	0.084	0.010	4.0 × 10^−15^	–	9.44%
				Adult women	948	0.271	0.257	0.058	0.007	1.7 × 10^−17^	–	6.35%
				***Combined analysis in validation dataset***	**4271**	**0.282**	**0.262**	**0.057**	**0.003**	**9.3 × 10**^**−83**^	**0.0458**	–
				**Adults**	**426**	**0.272**	**0.269**	**0.066**	**0.012**	**2.3 × 10**^**−8**^	–	5**.**58%
				T2D patients in HKDR study	4917	0.222	0.226	0.064	0.004	7.4 × 10^−64^	–	5.24%
				T2D patients in HKDB phase 1 study	1941	0.239	0.237	0.073	0.006	8.6 × 10^−32^	–	6.06%
				T2D patients in HKDB phase 2 study	865	0.267	0.252	0.077	0.008	6.1 × 10^−21^	–	8.27%
				***Combined analysis in T2D patients***	**7723**	**0.231**	**0.232**	**0.068**	**0.003**	**3.2 × 10**^**− 116**^	**0.1906**	–
PRS_LDL_	LDL-C	84	Pruning and thresholding	Children	909	0.239	0.243	0.068	0.008	1.1 × 10^−15^	–	6.81%
			(*r*^2^ = 0.2 and *p* = 5 × 10^−8^)	Adolescents	1973	0.309	0.294	0.093	0.006	2.6 × 10^−53^	–	10.88%
				Healthy adults	441	0.274	0.274	0.077	0.012	9.8 × 10^−10^	–	7.22%
				Adult women	948	0.262	0.275	0.071	0.008	1.0 × 10^−16^	–	6.33%
				***Combined analysis in validation dataset***	**4271**	**0.281**	**0.277**	**0.081**	**0.004**	**2.4 × 10**^**−93**^	**0.0439**	–
				**Adults**	**426**	**0.255**	**0.288**	**0.072**	**0.012**	**5.8 × 10**^**−9**^	–	6**.**72%
				T2D patients in HKDR study	4917	0.178	0.175	0.059	0.004	1.8 × 10^−38^	–	3.51%
				T2D patients in HKDB phase 1 study	1941	0.190	0.175	0.054	0.006	3.0 × 10^−17^	–	3.60%
				T2D patients in HKDB phase 2 study	865	0.195	0.171	0.058	0.010	9.2 × 10^−9^	–	3.74%
				***Combined analysis in T2D patients***	**7723**	**0.183**	**0.175**	**0.057**	**0.003**	**3.2 × 10**^**−62**^	**0.8303**	–

The optimal polygenic risk scores for four lipid traits were chosen based on the validation datasets which consisted of 909 children, 1973 adolescents, 441 health adults, and 948 adult women. These scores were further tested in 426 adults from the general population and 7723 individuals from three cohorts of T2D patients. Associations between polygenic risk scores and lipid traits were assessed by Pearson and Spearman correlations and a linear regression model. Within individual cohorts, *p* values were obtained from linear regression with adjustment of principal components, sex, age, and body mass index. Lipid traits were natural log-transformed for the linear regression analysis. Results from cohorts of children, adolescents, healthy adults, and adult women were meta-analyzed using a fixed effects model in the validation stage (*n* = 4271), while results from three cohorts of T2D patients were combined in the testing stage (*n* = 7723). *P*_*Q*_ refers to the *p* value of Cochran’s *Q*-statistics in heterogeneity test. The proportion of variance for a lipid trait explained by the corresponding optimal PRS was computed as the *R*^*2*^ obtained from a full model including both PRS and covariates (PCs, sex, age, and BMI) minus the *R*^*2*^ obtained from a model including covariates alone

We further tested the predictive capability of the four optimal PRSs on lipid traits in additional 426 adults from the general population and 7723 patients with T2D (See Table 1 and Additional File 1: Fig. S4). The Pearson correlations between these PRSs and the corresponding lipid measurements in the adults were generally comparable with the validation datasets, except for total cholesterol (0.251 < correlation coefficients (*r*) < 0.272). However, the pooled correlations were consistently lower in T2D patients compared with the validation datasets (0.185 vs 0.304 for TC; 0.206 vs 0.256 for TG; 0.231 vs 0.282 for HDL-C; and 0.183 vs 0.281 for LDL-C). Likewise, these PRSs explained only 3.0–3.9% of the variance for TC, 5.0–7.3% for TG, 5.2–8.3% for HDL-C, and 3.5–3.7% for LDL-C in T2D patients. With the adjustments for PCs sex, age, and BMI, there was an elevation of 4.0% in TC (*P* = 1.5 × 10^− 66^), 16.7% in TG (*P* = 2.3 × 10^− 126^), 7.0% in HDL-C (*P* = 3.2 × 10^− 116^), and 5.9% in LDL-C (*P* = 3.2 × 10^− 62^) per 1-SD increase in corresponding PRS in patients with T2D. The discrepancy between validation and testing datasets may reflect (1) the differences in characteristics of T2D patients and individuals of the general population and 2) some overfitting due to small sample size and different age groups in the validation datasets.

The best-performing PRSs for the four lipid traits built in the current study had considerably greater abilities to predict variation in plasma lipids than the four PRSs which comprised of only the 70–102 lead variants previously reaching genome-wide significance in European populations. The latter four PRSs had correlations of only 0.089 < *r* < 0.191 with the corresponding lipid traits in adults from the general population (See Additional File 2: Table S6). Although the correlations were markedly increased to 0.215–0.240 when the PRSs involved both the lead and independent variants, our four optimal PRSs still had better performance than these scores (See Additional File 2: Table S6). Similar results were also observed in patients with T2D, except the PRS for HDL-C (Additional File 2: Table S6).

### Predictive power of PRSs for identifying individuals with clinically defined dyslipidemia

We assessed the contribution of the lipid-specific PRSs for predicting the risk of developing dyslipidemia. AUC was used to assess the discriminatory power of the model with and without inclusion of PRS on top of clinical factors (sex, age, and BMI) and PCs. In the model incorporating the corresponding PRS alone, the AUCs for predicting abnormal levels of TC ≥ 5.2 mmol/l, TG ≥ 1.7 mmol/l, TG ≥ 1.97 mmol/l, and LDL ≥ 2.6 mmol/l varied between 0.63 and 0.67 in the general population; but was relatively lower in T2D patients, varying from 0.57 to 0.64 (See model 2 in Additional File 2: Table S7). We then examined whether the addition of these PRSs improved the risk prediction above and beyond traditional clinical risk factors. Risk assessment based on sex, age, BMI, PCs, and corresponding lipid-specific PRS significantly increased the AUC by 0.032–0.057 in the general population (7.5 × 10^− 3^ < *P* < 0.0400) and 0.029–0.069 in T2D patients (2.1 × 10^− 10^ < *P* < 0.0428), compared with the model incorporating the clinical factors and PCs only (See Additional File 2: Table S7). Interestingly, we further observed that the model incorporating the lipid-specific PRS alone had higher prediction accuracy for abnormal levels of TC and LDL-C than the model involving the clinical risk factors and PCs only in children and adolescents (See model 1 vs model 2 in Additional File 2: Table S8).

We then evaluated the precision of the high PRSs for diagnosing dyslipidemia (See Additional File 2: Tables S7 and S8). For example, individuals who carry a high PRS (the top 20% of the PRS distribution) for TC had a positive predictive value (PPV) of 69.2% in the general population and 31.5–62.7% in T2D patients. Negative predictive values (NPV) were 57.1% and 53.1–76.3%, respectively.

### Impact of PRSs on 3-year changes in lipid levels in adolescents

In this analysis, we included 620 adolescents with lipid profiles measured at baseline and during follow-up (See Additional File 2: Table S9). As expected, we found strong relationships between all four PRSs and their corresponding lipid measurements at baseline (7.5 × 10^− 16^ < *P* < 9.6 × 10^− 13^) and during follow-up (6.7 × 10^− 17^ < *P* < 5.5 × 10^− 8^) among the subset of adolescents (See Table 2 and Additional File 1: Fig. S5). Interestingly, we observed that these PRSs were in addition also associated with the 3-year changes in corresponding lipid levels, after accounting for the baseline measurements (1.4 × 10^− 6^ < *P* < 0.0130) (See Table 2 and Additional File 1: Fig. S5).

**Table 2:** Association between polygenic risk scores and longitudinal changes in lipid levels over 3 years in adolescents (*n* = 620)

Polygenic risk score	Lipid trait	Lipid traits at baseline (***n*** = 620)			Lipid traits at follow-up (***n*** = 620)			Three-year changes in lipid traits (***n*** = 620)		
		***β***	SE	***P***^***a***^	***β***	SE	***P***^***b***^	***β***	SE	***P***^***c***^
PRS_TC_	Total cholesterol	0.0507	0.0066	6.3 × 10^−14^	0.0480	0.0064	2.7 × 10^−13^	0.0151	0.0050	2.8 × 10^−3^
PRS_TG_	Triglycerides	0.1076	0.0146	5.9 × 10^−13^	0.0843	0.0153	5.5 × 10^−8^	0.0361	0.0145	0.0130
PRS_HDL_	HDL cholesterol	0.0496	0.0068	9.6 × 10^−13^	0.0615	0.0071	6.7 × 10^−17^	0.0285	0.0058	1.4 × 10^−6^
PRS_LDL_	LDL cholesterol	0.0863	0.0104	7.5 × 10^−16^	0.0849	0.0106	4.7 × 10^−15^	0.0236	0.0080	3.1 × 10^− 3^

Lipid traits at baseline and follow-up were natural log (*ln*) transformed. The 3-year changes in lipid traits were transformed as *ln*(Y + 1). ^a^ *P* values were obtained from linear regression with the adjustment for principal components, sex, age at baseline, and BMI at baseline. ^b^ *P* values were obtained from linear regression with the adjustment for principal components, sex, age at follow-up, and BMI at baseline and follow-up. ^c^ *P* values were obtained from linear regression with the adjustment for principal components, sex, age at follow-up, BMI at baseline and follow-up, and lipid trait at baseline

### Association between PRSs and carotid intima-media thickness (cIMT) in adult women

To explore the polygenic susceptibility to subclinical atherosclerosis, we stratified the PRS for each lipid trait into five categories according to the quintiles in two independent cohorts of adult women and performed a linear regression in each cohort, followed by a meta-analysis to find its association with cIMT in two different ways. First, we examined a linear trend across the quintile categories. Second, we tested a hypothesis that a high PRS for TC, TG, and LDL-C (a low PRS for HDL-C) was associated with cIMT by comparing the top (bottom) 20% with the remaining 80% of the PRS distribution. Descriptive statistics for the 2 cohorts of adult women are provided in Additional File 2: Table S10. Independent of PCs and age, the best PRS for TC had a positive but modest linear relationship with cIMT in meta-analysis (*P* = 0.0182; see model 1 in Table 3). Further inclusion of BMI and systolic blood pressure (SBP) as covariates minimally affected this result (*P* = 0.0315; see model 2 in Table 3).

**Table 3:** Association between quintiles of polygenic risk scores and carotid intima-media thickness in adult women (*n* = 781)

Polygenic risk score	Cohorts of adult women	Quintile of polygenic score						Model 1		Model 2	
		Q1	Q2	Q3	Q4	Q5	Test	*P*_model 1_	*P*_*Q*_	*P*_model 2_	*P*_*Q*_
PRS_TC_	Cohort 1	0.0481 (0.0466–0.0497)	0.0496 (0.0482–0.0511)	0.0503 (0.0487–0.0520)	0.0501 (0.0486–0.0516)	0.0497 (0.0482–0.0514)	Linear trend	0.0534		0.1003	
	Cohort 2	0.0481 (0.0450–0.0515)	0.0504 (0.0453–0.0561)	0.0529 (0.0479–0.0584)	0.0550 (0.0496–0.0610)	0.0512 (0.0479–0.0547)	Linear trend	0.1032		0.0758	
							**Meta-analysis**	**0.0182**	**0.3547**	**0.0315**	**0.2560**
	Cohort 1	0.0495 (0.0488–0.0503)				0.0497 (0.0482–0.0514)	Top 20% vs others	0.6893		0.7445	
	Cohort 2	0.0518 (0.0493–0.0544)				0.0512 (0.0479–0.0547)	Top 20% vs others	0.8236		0.9732	
							**Meta-analysis**	**0.7591**	**0.7336**	**0.7667**	**0.8891**
PRS_TG_	Cohort 1	0.0499 (0.0485–0.0514)	0.0498 (0.0481–0.0515)	0.0491 (0.0475–0.0507)	0.0494 (0.0481–0.0507)	0.0498 (0.0480–0.0516)	Linear trend	0.7416		0.7173	
	Cohort 2	0.0515 (0.0471–0.0563)	0.0514 (0.0481–0.0548)	0.0512 (0.0477–0.0549)	0.0542 (0.0457–0.0643)	0.0507 (0.0469–0.0548)	Linear trend	0.6197		0.9116	
							**Meta-analysis**	**0.6430**	**0.7068**	**0.7048**	**0.9963**
	Cohort 1	0.0495 (0.0488–0.0503)				0.0498 (0.0480–0.0516)	Top 20% vs others	0.9536		0.9879	
	Cohort 2	0.0518 (0.0495–0.0542)				0.0507 (0.0469–0.0548)	Top 20% vs others	0.4509		0.9879	
							**Meta-analysis**	**0.8786**	**0.4574**	**0.9917**	**0.9849**
PRS_HDL_	Cohort 1	0.0512 (0.0497–0.0527)	0.0490 (0.0475–0.0505)	0.0496 (0.0479–0.0513)	0.0483 (0.0469–0.0498)	0.0498 (0.0482–0.0514)	Linear trend	0.3786		0.3242	
	Cohort 2	0.0511 (0.0470–0.0556)	0.0542 (0.0466–0.0630)	0.0498 (0.0459–0.0539)	0.0532 (0.0491–0.0576)	0.0508 (0.0475–0.0542)	Linear trend	0.9307		0.8528	
							**Meta-analysis**	**0.4194**	**0.7165**	**0.3205**	**0.8838**
	Cohort 1	0.0512 (0.0497–0.0527)	0.0492 (0.0484–0.0500)				Bottom 20% vs others	0.0212		0.0160	
	Cohort 2	0.0511 (0.0470–0.0556)	0.0518 (0.0495–0.0542)				Bottom 20% vs others	0.4012		0.4524	
							**Meta-analysis**	**0.0533**	**0.1286**	**0.0405**	**0.1373**
PRS_LDL_	Cohort 1	0.0481 (0.0467–0.0495)	0.0498 (0.0484–0.0513)	0.0489 (0.0475–0.0503)	0.0514 (0.0494–0.0536)	0.0495 (0.0482–0.0509)	Linear trend	0.0485		0.0954	
	Cohort 2	0.0509 (0.0452–0.0574)	0.0516 (0.0458–0.0581)	0.0533 (0.0485–0.0585)	0.0545 (0.0504–0.0590)	0.0490 (0.0465–0.0517)	Linear trend	0.9030		0.7916	
							**Meta-analysis**	**0.0570**	**0.5840**	**0.0972**	**0.7430**
	Cohort 1	0.0496 (0.0488–0.0504)				0.0495 (0.0482–0.0509)	Top 20% vs others	0.7463		0.7438	
	Cohort 2	0.0526 (0.0500–0.0553)				0.0490 (0.0465–0.0517)	Top 20% vs others	0.2775		0.3636	
							**Meta-analysis**	**0.9340**	**0.2564**	**0.9800**	**0.3328**

Data are presented as geometric mean (95% CI) stratified by quintile categories of polygenic risk score. Intima-media thickness was natural log (*ln*) transformed. *P*_*model 1*_ and *P*_*model 2*_ values were obtained from linear regression with the adjustment for covariates included in models 1 and 2, respectively. Model 1: principal components and age. Model 2: principal components, age, body mass index, and systolic blood pressure. Results from individual cohorts were meta-analyzed using a fixed effects model. *P*_*Q*_ refers to the *p* value of Cochran’s *Q*-statistics in heterogeneity test. Associations between PRSs and carotid intima-media thickness were examined in two different ways: (1) we examined a linear trend across the quintile categories. (2) We tested a hypothesis that a high PRS for TC, TG, and LDL-C (a low PRS for HDL-C) was associated with intima media thickness by comparing the top (bottom) 20% with the remaining 80% of the PRS distribution

### The risk of CHD according to the quintile of PRSs in patients with T2D

**Fig. 2:**
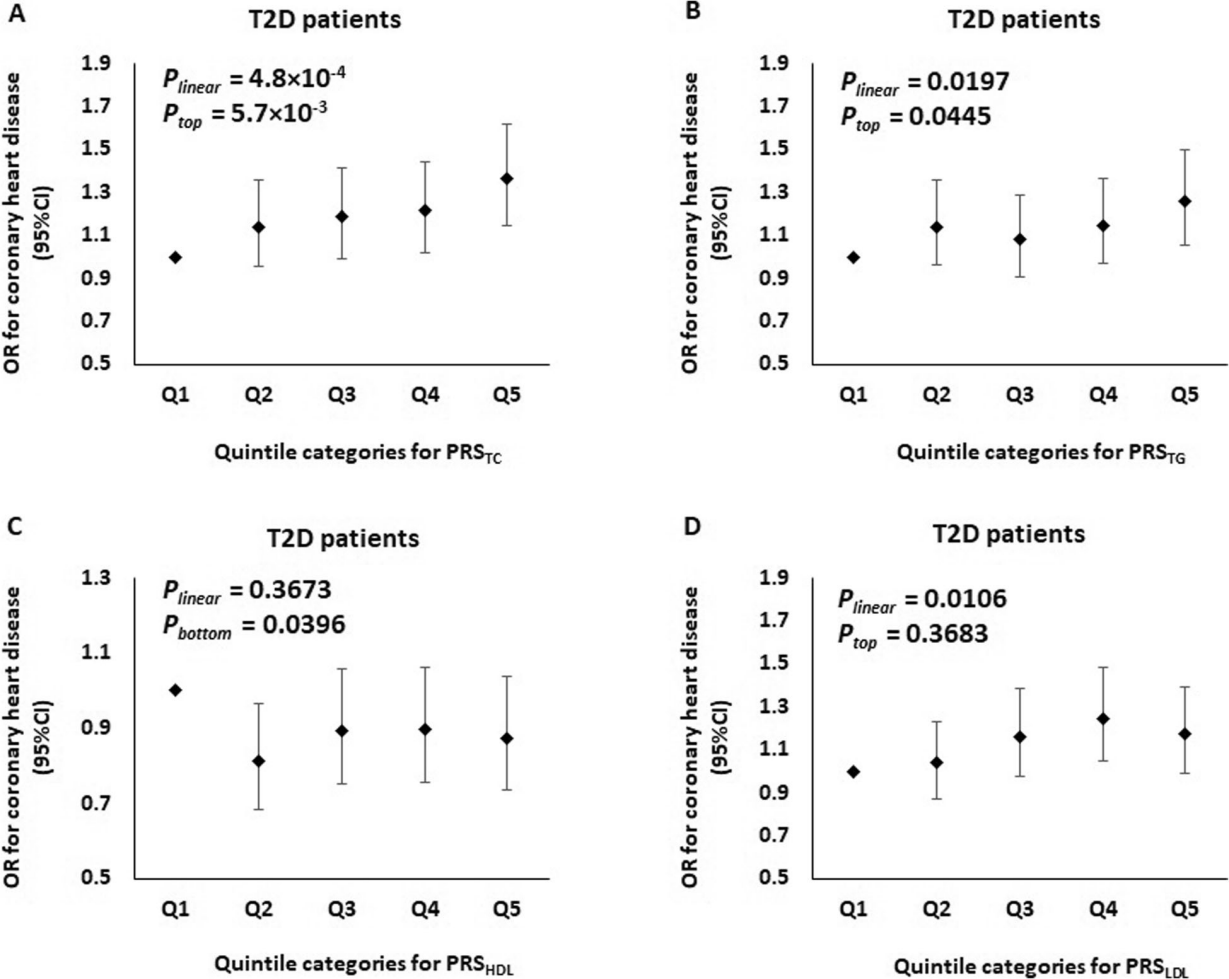
Odds ratio (OR) of coronary heart disease stratified by quintile of polygenic risk scores [**a** PRS_TC_, **b** PRS_TG_, **c** PRS_HDL_, and **d** PRS_LDL_] in T2D patients (*n* = 2374 cases vs 6246 controls). *P*_linear_ refers to the *p* value testing for a linear trend across five quintiles of polygenic risk score. *P*_top_ refers to the *p* value testing for the association of a high polygenic risk score with coronary heart disease by comparing the top 20% of the distribution with the remaining 80% of the distribution. *P*_bottom_ refers to the *p* value testing for the association of a low polygenic risk score with coronary heart disease by comparing the bottom 20% of the distribution with the remaining 80% of the distribution. Within each individual cohort, all *p* values were obtained from logistic regression with the adjustment of principal components, sex, age, duration of diabetes, body mass index, smoking status, HbA1c, systolic blood pressure, estimated glomerular filtration rate, and log-transformed albumin-creatinine ratio. Results from individual cohorts were meta-analyzed using fixed effects model

Next, we evaluated the role of four PRSs for lipid traits in predicting the risk of CHD in two prospective cohorts of T2D patients (total *n* = 2374 CHD cases and 6246 controls). Clinical characteristics of these patients are summarized in Additional File 2: Table S11. With adjustments for PCs, sex, age, and duration of diabetes, the best-performing PRSs for TC, TG, and LDL-C were significantly but moderately associated with increased risk for CHD in patients with T2D (2.7 × 10^− 3^ < *P* < 0.0219) (See model 1 in Additional File 2: Table S12). These associations were also independent of other covariates, including BMI in model 2, smoking status in model 3, metabolic risk factors (HbA_1c_ level and SBP) in model 4, and renal function (eGFR and log-transformed ACR) in model 5 (*P* < 0.05) (See models 2–5 in Additional file 2: Table S12, and Fig. 2). We found that going up each quintile of these PRSs raised the odds of CHD by approximately 5–7% (4.8 × 10^− 4^ < *P* < 0.0197) (See model 5 in Additional file 2: Table S12, and Fig. 2). On the other hand, we further observed that for these PRSs, patients with diabetes who had a high (top quintile) PRSs for TC or TG resulted in increasing risk of CHD by 15–20% (5.7 × 10^− 3^ < *P* < 0.0445) (See model 5 in Additional file 2: Table S12, and Fig. 2). However, these associations were markedly attenuated when we further adjusted for the use of lipid-lowering medications at baseline (See model 6 in Additional file 2: Table S12).

## Discussion

Leveraging on the association statistics from the BBJ project and the individual-level data from multiple Chinese cohorts at various stages of the life-course, we applied recently developed computational methods to construct four novel East Asians-specific PRSs, which aggregate genetic information from 84 to 549 common SNPs. These PRSs were then used to identify individuals at high risk of dyslipidemia. We also found associations of lipid-specific PRSs with longitudinal changes in lipid levels over 3 years, subclinical atherosclerosis, and diabetes cardiovascular complications.

It remains largely unknown how genetic factors influence changes in lipid levels across one’s lifetime. Using longitudinal data in 620 adolescents, we have computed the average changes in lipid levels to summarize both the direction and magnitude of changes in lipids over a 3-year period. This study reveals that aggregation of common genetic variants which were selected using a liberal *p* value threshold for variant inclusion, while accounting for LD patterns in the East Asian population, provided independent information to predict dyslipidemia and longitudinal changes in lipids over 3 years, beyond other established risk factors such as sex, age, and BMI. Although the determinants of lipid levels and developmental trajectories are multifactorial, our PRSs are highly predictive for the corresponding lipid measurements at different stages of the life-course. More importantly, the lipid-specific PRSs are better at predicting abnormal levels of TC and LDL-C than the typical risk factors at younger age. A few studies have prospectively evaluated the lipid profiles, and their observations paralleled those herein obtained. For instance, a longitudinal analysis of cardiovascular risk in a study of young Finns assessed the association of GWAS-derived PRSs with TG, HDL-C, and LDL-C trajectories from childhood to adulthood in 2442 participants [35]. In support of our findings, the authors demonstrated the significance of PRSs as predictors of lipid levels at all ages; however, no clear divergence of lipid trajectories over time between PRS categories was found. Recently, Lu et al. conducted a GWAS of blood lipid levels including more than twenty thousand individuals from Han Chinese ancestry [36]. In a subset prospective cohort of 6428 adults with > 8.1 years of follow-up, they reported that the four lipid-related PRSs were independently associated with linear increases in their corresponding lipid levels and risk of incident hyperlipidemia. Their C-statistics analysis further revealed significant improvement in the prediction of incident hyperlipidemia beyond conventional risk factors including the baseline lipid levels (1–2% increases in C-statistics). Taken altogether, these findings suggest that PRSs can provide a robust prediction for average lipid levels and lipid changes across a person’s lifetime. These findings also highlight the influence of genetics on lipid variation in early life. In contrast to most of the conventional risk factors, genetic information can be measured at an early age. It may play a role in disease risk prediction when clinical risk factors have yet to manifest.

Variant selection is one of the challenges in the construction of PRS. Compared with typical PRSs based on genome-wide significant variants, our results showed that addition of less-significant SNPs in the computation of PRS consistently improved the polygenic risk prediction for some lipid levels across different age groups in the Chinese population, although decreases in performance were noted in T2D patients. These findings also highlight the need for ethnic−/population-specific PRSs. In the context of the overwhelming abundance of GWAS in European populations, PRSs for complex traits and diseases have predominantly been derived and tested in European populations. Nevertheless, it has been suggested that PRSs do not transfer well between ancestral groups [37]. Previous studies demonstrated generally lower predictive power of European ancestry-derived PRSs in non-European ancestry individuals, supporting the observation of our current study [38]. For example, the Million Veteran Program, which consists of ~ 300 K individuals in which > 70% are Caucasians, recently reported that a total of 826 independent lipid variants explained about 8.8–12.3% of the phenotypic variance in lipid levels, comparatively higher than that observed in this study (See Additional file 2: Table S6) [2]. These findings highlighted issues regarding ethnic-based SNP bias, whereby certain genetic variants may not have the same phenotypic effects in different ancestral populations [39]. GWAS favor the identification of common genetic variants in the discovery population. Differences in LD and variant allele frequencies across populations might impact the heritability for the same phenotype in other populations (e.g., low-frequency variants display larger average effects on phenotype compared with common variants). We further noted that even though the current PRSs for lipid traits were developed and validated in populations of East Asian descent, they consistently predict the lipid levels far more accurately in the general population (validation datasets) than among subjects with T2D (testing datasets), regardless of the choice of computational algorithms and the type of lipids (See Additional File 1: Fig. S2, and Additional File 2: Tables S13 – S16). In fact, many factors, including environmental factors, may differ across populations within the same ancestry, and thereby modify effects such as gene-environment interaction, leading to problems of comparability across diverse human populations [40]. Because of limited studies in non-European populations, this study highlighted the possibility of constructing custom PRSs in other specific populations, such as East Asians where there are accumulating GWAS, to facilitate the development of precision medicine. However, there is a need for further large-scale GWAS or meta-analyses in these non-European populations.

Although PRSs are likely to be ethnic-specific, their utility has been confirmed in different populations [38]. In addition to multiple studies demonstrating the ability of PRS to predict dyslipidemia or cardiovascular diseases (CVD), emerging insights suggest that individuals at extreme ends of the risk continuum, according to inheritance of common variants, have disease risk that may be comparable to individuals carrying monogenic gene mutations. For example, a study of genome-wide PRSs by Khera et al. found that 8% of European ancestry individuals in the UK Biobank have a PRS-defined risk of coronary artery disease risk that was comparable or higher than those who harbor rare Familial Hyercholesterolemia mutations [7]. Another study of 53 Finnish families of familial combined hyperlipidemia (FCH) showed that approximately a third of the affected FCH individuals had high polygenic burden (the top 10% of the PRS distribution), which is comparable to that observed in individuals with similar lipid levels in the general population [41]. Therefore, individuals at the tails of the risk distribution may potentially be targeted for intensive treatments to lower CHD risk.

Among T2D patients, we found at least marginally significant associations of PRSs for TC, TG, and LDL-C with CHD risk, independent of established clinical risk factors. The TC-related PRS was also moderately associated with cIMT in adult women, supporting the observed association for CHD in patients with T2D. Although we have not specifically investigated the association between our new PRSs and risk of CVD in a general population, numerous studies have confirmed the association of PRS that capture the overall genetic risk of lipid traits, with subclinical atherosclerosis and cardiovascular outcomes [4, 5, 42, 43]. In a study of 10,399 Europeans drawn from the Erasmus Rucphen Family Study and the Rotterdam Study, the accumulation of 32–52 common SNPs with small effects on four lipid levels was significantly associated with carotid plaque, a surrogate marker of cardiovascular disease [5]. Similar to our findings, both TC and LDL-C risk scores were nominally associated with elevated cIMT (increased 0.004–0.006 mm per SD increase in score) and increased risk of CHD (hazard ratio, 1.08–1.10 per SD increase in score) [5]. Recently, further studies have utilized Mendelian randomization (MR) approaches to examine the causal roles of TG, HDL-C, and LDL-C in CHD [4, 42, 44–46]. Holmes et al. developed two kinds of PRSs based on SNPs with established associations with TG, HDL-C, and LDL-C and performed MR meta-analyses in 62,199 participants with 12,099 CHD events [42]. The unrestricted PRSs included all independent SNPs each associated with a specific lipid trait identified from a prior meta-analysis; and the restricted PRSs excluded any SNPs also associated with either of the other two lipid traits. Their MR analyses showed that a genetically elevated LDL-C and TG, regardless of the types of PRSs, resulted in an increased causal odds ratio (OR) for CHD risk. The causal OR of LDLs is similar in magnitude to that reported in randomized trials of statin-lowering therapies in individuals at low risk of vascular disease [47]. The MR analysis further demonstrated the causal role of LDL-C in cIMT, supporting the use of cIMT as an appropriate surrogate marker of therapies that modulate LDL-C. However, several previous MR analysis using different genetic instruments failed to identify a clear causal role of HDL-C in CHD [42, 46]. Few studies so far have examined the utility of PRS generated from the general population in subjects with T2D. Our overall findings, despite modest sample sizes, suggest a population-specific PRS for LDL-C and TG also identifies increased risk of CHD among subjects with T2D, consistent with these findings. In addition, we noted higher risks of CHD in T2D patients with higher genetic risk for dyslipidemia (e.g., individuals in the top 20% of the PRSs for TC and TG). This association was substantially attenuated by the adjustment of baseline lipid-lowering therapies, suggesting that individuals at high genetic risk may derive the greatest benefit from early intervention to reduce CVD. Indeed, non-prescribing and non-adherence are common in real-world practice [48]. Our PRSs can be considered as candidates to motivate behavioral changes such as drug adherence.

There are several limitations in this study. First, our PRSs were derived and tested in individuals of East Asians descent only, with limited generalizability. Because of the discrepancy in genomic structure, culture, and environmental factors, as well as potential differences in phenotypic effect of genes across ethnicities, these East Asians-specific PRSs might not have optimal predictive power in other ethnic groups. Second, only common genetic variants with MAF > 1% were included in the PRSs in the current study. The addition of low-frequency variants, gene-gene, and gene-environmental interaction to current PRSs would enable more precise prediction. Third, we acknowledged that our multiple study cohorts with a relatively small sample size may not be able to accurately and comprehensively estimate both the phenotypic variation and the genetic diversity in our population. In fact, several genome-wide PRSs for lipid traits comprised of millions of SNPs have been derived in the non-Asian populations and demonstrated to perform better than more limited scores [49, 50]. One explanation for the modest number of SNPs in our PRSs is that the total level of genetic variation covered in our validation datasets is less than that in previous larger studies. Therefore, fewer SNPs were required to differentiate the genetic diversity in the current study. Fourth, we assigned the weight to each unfavorable allele in current PRSs for lipid traits based on its contribution to the corresponding lipid levels. The effect of each genetic variant on subclinical atherosclerosis and the risk of CHD might not be linearly related to its effects on lipid traits. Furthermore, because of the comparatively short duration of follow-up in the HKDB study (i.e., 2 years), patients might develop CHD in the future.

## Conclusions

We have applied a systematic approach to derive and validate four PRSs for lipid traits in the East Asian population. These PRSs were strongly associated with their corresponding measured lipid levels and longitudinal changes in lipid levels over 3 years, which began to emerge in childhood and adolescence, though there was reduced association in T2D patients. Independent of conventional risk factors, patients with a higher genetic susceptibility to dyslipidemia had an increased risk for CHD. Further adjustment for lipid drug use notably attenuated this association. We also found a modest association of TC-related PRSs with subclinical atherosclerosis (e.g., cIMT) in adult women. Altogether, this study highlights the potential utility of polygenic risk predictors in clinical therapy as they facilitate the identification of at-risk individuals from early life, before the presence of clinical manifestation, which may help to empower earlier intervention among at-risk individuals. To provide best performance, PRSs specific for diverse human populations may be required.

## Supplementary Information

**Additional file 1:** Supplementary methods: 1) Hong Kong Diabetes Register TRS Study Group Members; 2) Hong Kong Diabetes Biobank Study Group Members; and 3) Cohort descriptions. **Figure S1.** Principal component analysis (PCA). **Figure S2.** Pooled correlations of each candidate polygenic risk scores with measured lipid traits. **Figure S3.** Proportion of phenotypic variance in lipid traits explained by each candidate polygenic risk scores. **Figure S4.** Geometric means of measured lipid traits stratified by the quintile of polygenic risk score with the best performance. **Figure S5.** Geometric means of measured lipid traits at baseline and follow-up, and three-year changes in lipid traits stratified by quintile of polygenic risk scores in adolescents.

**Additional file 2: Table S1.** Clinical characteristics of all participants. **Table S2.** Correlations of candidate polygenic risk scores with total cholesterol in validation datasets. **Table S3.** Correlations of candidate polygenic risk scores with triglyceride levels in validation datasets. **Table S4.** Correlations of candidate polygenic risk scores with HDL cholesterol in validation datasets. **Table S5.** Correlations of candidate polygenic risk scores with LDL cholesterol in validation datasets. **Table S6.** Correlations between measured lipid traits and polygenic risk scores derived by using the genome-wide significant variants identified in European populations. **Table S7.** Prediction ability of the best polygenic risk scores for abnormal lipid levels in testing datasets. **Table S8.** Prediction ability of the best polygenic risk scores for abnormal lipid levels in validation datasets. **Table S9.** Baseline and follow-up clinical characteristics of the adolescents included in the assessment of three-year changes for lipid traits. **Table S10.** Clinical characteristics of the adult women included in the assessment of intima-media thickness. **Table S11.** Clinical characteristics of the T2D patients included in the assessment of coronary heart disease. **Table S12.** Association between coronary heart disease and quintiles of polygenic risk scores in T2D patients. **Table S13.** Correlations of candidate polygenic risk scores with total cholesterol in T2D patients. **Table S14.** Correlations of candidate polygenic risk scores with triglyceride levels in T2D patients. **Table S15.** Correlations of candidate polygenic risk scores with HDL cholesterol in T2D patients. **Table S16.** Correlations of candidate polygenic risk scores with LDL cholesterol in T2D patients. **Table S17.** Baseline clinical characteristics of the adolescents stratified by the status of follow-up.

## Data Availability

The PRSs reported in this manuscript have been deposited into the Polygenic Score (PGS) catalog (https://www.pgscatalog.org/). The respective PGS ID numbers are listed below: PRS-TC PGS000658 www.pgscatalog.org/score/PGS000658 PRS-TG PGS000659 www.pgscatalog.org/score/PGS000659 PRS-HDL PGS000660 www.pgscatalog.org/score/PGS000660 PRS-LDL PGS000661 www.pgscatalog.org/score/PGS000661 Publication PGP000121 www.pgscatalog.org/publication/PGP000121/ The authors declare that the data supporting the findings of this study are available within the manuscript and its additional files. The individual-level data of this study are available on request from the corresponding author (R.C.W.M). The data are not publicly available since they contain information that could compromise research participant privacy/consent.

## Abbreviations

TC: Total cholesterol
TG: Triglycerides
HDL-C: High-density lipoprotein cholesterol
LDL-C: Low-density lipoprotein cholesterol
CHD: Coronary heart disease
GWAS: Genome-wide association study
PRS: Polygenic risk score
LD: Linkage disequilibrium
T2D: Type 2 diabetes
BBJ: BioBank Japan
HAPO: Hyperglycemia and Adverse Pregnancy Outcome
HKDR: Hong Kong Diabetes Register
HKDB: Hong Kong Diabetes Biobank
cIMT: Carotid intima-media thickness
QC: Quality control
SNP: Single nucleotide polymorphism
IBD: Identity-by-descent
PC: Principal component
HWE: Hardy–Weinberg equilibrium
MAF: Minor allele frequency
BMI: Body mass index
SBP: Systolic blood pressure
eGFR: Estimated glomerular filtration rate
ACR: Albumin-creatinine ratio
ROC: Receiver operating characteristic
AUC: Area under curve
1-SD: One standard deviation
MR: Mendelian randomization

## References

[1] Weiss LA, Pan L, Abney M, Ober C (2006). The sex-specific genetic architecture of quantitative traits in humans. Nat Genet.

[2] Klarin D, Damrauer SM, Cho K, Sun YV, Teslovich TM, Honerlaw J (2018). Genetics of blood lipids among ~300,000 multi-ethnic participants of the Million Veteran Program. Nat Genet.

[3] Natarajan P (2018). Polygenic risk scoring for coronary heart disease: the first risk factor. J Am Coll Cardiol.

[4] Kathiresan S, Melander O, Anevski D, Guiducci C, Burtt NP, Roos C (2008). Polymorphisms associated with cholesterol and risk of cardiovascular events. N Engl J Med.

[5] Isaacs A, Willems SM, Bos D, Dehghan A, Hofman A, Ikram MA (2013). Risk scores of common genetic variants for lipid levels influence atherosclerosis and incident coronary heart disease. Arterioscler Thromb Vasc Biol.

[6] Inouye M, Abraham G, Nelson CP, Wood AM, Sweeting MJ, Dudbridge F (2018). Genomic risk prediction of coronary artery disease in 480,000 adults: implications for primary prevention. J Am Coll Cardiol.

[7] Khera AV, Chaffin M, Aragam KG, Haas ME, Roselli C, Choi SH (2018). Genome-wide polygenic scores for common diseases identify individuals with risk equivalent to monogenic mutations. Nat Genet.

[8] Khera AV, Chaffin M, Wade KH, Zahid S, Brancale J, Xia R (2019). Polygenic prediction of weight and obesity trajectories from birth to adulthood. Cell..

[9] Kanai M, Akiyama M, Takahashi A, Matoba N, Momozawa Y, Ikeda M (2018). Genetic analysis of quantitative traits in the Japanese population links cell types to complex human diseases. Nat Genet.

[10] Nagai A, Hirata M, Kamatani Y, Muto K, Matsuda K, Kiyohara Y (2017). Overview of the BioBank Japan Project: study design and profile. J Epidemiol.

[11] Tam WH, Ma RCW, Ozaki R, Li AM, Chan MHM, Yuen LY (2017). In utero exposure to maternal hyperglycemia increases childhood cardiometabolic risk in offspring. Diabetes Care.

[12] Ozaki R, Qiao Q, Wong GW, Chan MH, So WY, Tong PC (2007). Overweight, family history of diabetes and attending schools of lower academic grading are independent predictors for metabolic syndrome in Hong Kong Chinese adolescents. Arch Dis Child.

[13] Ko GT, Chan JC, Chan AW, Wong PT, Hui SS, Tong SD (2007). Association between sleeping hours, working hours and obesity in Hong Kong Chinese: the 'better health for better Hong Kong' health promotion campaign. Int J Obes.

[14] Hu M, Yang YL, Chan P, Tomlinson B (2015). Pharmacogenetics of cutaneous flushing response to niacin/laropiprant combination in Hong Kong Chinese patients with dyslipidemia. Pharmacogenomics..

[15] Jiang G, Luk AOY, Tam CHT, Xie F, Carstensen B, Lau ESH (2019). Progression of diabetic kidney disease and trajectory of kidney function decline in Chinese patients with type 2 diabetes. Kidney Int.

[16] Jiang G, Luk AO, Tam CHT, Lau ES, Ozaki R, Chow EYK (2020). Obesity, clinical, and genetic predictors for glycemic progression in Chinese patients with type 2 diabetes: a cohort study using the Hong Kong Diabetes Register and Hong Kong Diabetes Biobank. PLoS Med.

[17] Tam WH, Ma RCW, Ozaki R, Li AM, Chan MHM, Yuen LY, et al. Antenatal treatment of gestational diabetes and offspring’s future cardiometabolic risk. the 9th International Symposium on Diabetes, Hypertension and Metabolic Syndrome and in Pregnancy; 8–12 March, 2017; Barcelona, Spain2017.

[18] Friedewald WT, Fredrickson DS, Levy RI (1972). Estimation of concentration of low-density lipoprotein cholesterol in plasma, without use of preparative ultracentrifuge. Clin Chem.

[19] Grundy SM, Stone NJ, Bailey AL, Beam C, Birtcher KK, Blumenthal RS (2019). 2018 AHA/ACC/AACVPR/AAPA/ABC/ACPM/ADA/AGS/APhA/ASPC/NLA/PCNA guideline on the management of blood cholesterol: a report of the American College of Cardiology/American Heart Association Task Force on Clinical Practice Guidelines. J Am Coll Cardiol.

[20] Liu KH, Chan YL, Chan JC, Chan WB (2005). Association of carotid intima-media thickness with mesenteric, preperitoneal and subcutaneous fat thickness. Atherosclerosis..

[21] Howie B, Fuchsberger C, Stephens M, Marchini J, Abecasis GR (2012). Fast and accurate genotype imputation in genome-wide association studies through pre-phasing. Nat Genet.

[22] Das S, Forer L, Schonherr S, Sidore C, Locke AE, Kwong A (2016). Next-generation genotype imputation service and methods. Nat Genet.

[23] Genomes Project C, Auton A, Brooks LD, Durbin RM, Garrison EP, Kang HM (2015). A global reference for human genetic variation. Nature..

[24] Chang CC, Chow CC, Tellier LC, Vattikuti S, Purcell SM, Lee JJ (2015). Second-generation PLINK: rising to the challenge of larger and richer datasets. Gigascience..

[25] Vilhjalmsson BJ, Yang J, Finucane HK, Gusev A, Lindstrom S, Ripke S (2015). Modeling linkage disequilibrium increases accuracy of polygenic risk scores. Am J Hum Genet.

[26] Chasman DI, Pare G, Mora S, Hopewell JC, Peloso G, Clarke R (2009). Forty-three loci associated with plasma lipoprotein size, concentration, and cholesterol content in genome-wide analysis. PLoS Genet.

[27] Teslovich TM, Musunuru K, Smith AV, Edmondson AC, Stylianou IM, Koseki M (2010). Biological, clinical and population relevance of 95 loci for blood lipids. Nature..

[28] Asselbergs FW, Guo Y, van Iperen EP, Sivapalaratnam S, Tragante V, Lanktree MB (2012). Large-scale gene-centric meta-analysis across 32 studies identifies multiple lipid loci. Am J Hum Genet.

[29] Albrechtsen A, Grarup N, Li Y, Sparso T, Tian G, Cao H (2013). Exome sequencing-driven discovery of coding polymorphisms associated with common metabolic phenotypes. Diabetologia..

[30] Willer CJ, Schmidt EM, Sengupta S, Peloso GM, Gustafsson S, Kanoni S (2013). Discovery and refinement of loci associated with lipid levels. Nat Genet.

[31] Peloso GM, Auer PL, Bis JC, Voorman A, Morrison AC, Stitziel NO (2014). Association of low-frequency and rare coding-sequence variants with blood lipids and coronary heart disease in 56,000 whites and blacks. Am J Hum Genet.

[32] Liu DJ, Peloso GM, Yu H, Butterworth AS, Wang X, Mahajan A (2017). Exome-wide association study of plasma lipids in >300,000 individuals. Nat Genet.

[33] Fisher RA (1915). Frequency distribution of the values of the correlation coefficient in samples from an indefinitely large population. Biometrika..

[34] DeLong ER, DeLong DM, Clarke-Pearson DL (1988). Comparing the areas under two or more correlated receiver operating characteristic curves: a nonparametric approach. Biometrics..

[35] Buscot MJ, Magnussen CG, Juonala M, Pitkanen N, Lehtimaki T, Viikari JS (2016). The combined effect of common genetic risk variants on circulating lipoproteins is evident in childhood: a longitudinal analysis of the cardiovascular risk in young Finns study. PLoS One.

[36] Lu X, Huang J, Mo Z, He J, Wang L, Yang X (2016). Genetic susceptibility to lipid levels and lipid change over time and risk of incident hyperlipidemia in Chinese populations. Circ Cardiovasc Genet.

[37] Reisberg S, Iljasenko T, Lall K, Fischer K, Vilo J (2017). Comparing distributions of polygenic risk scores of type 2 diabetes and coronary heart disease within different populations. PLoS One.

[38] Duncan L, Shen H, Gelaye B, Meijsen J, Ressler K, Feldman M (2019). Analysis of polygenic risk score usage and performance in diverse human populations. Nat Commun.

[39] Dron JS, Hegele RA (2019). The evolution of genetic-based risk scores for lipids and cardiovascular disease. Curr Opin Lipidol.

[40] Martin AR, Kanai M, Kamatani Y, Okada Y, Neale BM, Daly MJ (2019). Clinical use of current polygenic risk scores may exacerbate health disparities. Nat Genet.

[41] Ripatti P, Ramo JT, Soderlund S, Surakka I, Matikainen N, Pirinen M (2016). The contribution of GWAS loci in familial dyslipidemias. PLoS Genet.

[42] Holmes MV, Asselbergs FW, Palmer TM, Drenos F, Lanktree MB, Nelson CP (2015). Mendelian randomization of blood lipids for coronary heart disease. Eur Heart J.

[43] Trinder M, Francis GA, Brunham LR. Association of monogenic vs polygenic hypercholesterolemia with risk of atherosclerotic cardiovascular disease. JAMA Cardiol. 2020;5(4):390–9. 10.1001/jamacardio.2019.5954 PMC7042820 32049305

[44] Emerging Risk Factors C, Sarwar N, Sandhu MS, Ricketts SL, Butterworth AS, Triglyceride Coronary Disease Genetics C (2010). Triglyceride-mediated pathways and coronary disease: collaborative analysis of 101 studies. Lancet.

[45] Ference BA, Yoo W, Alesh I, Mahajan N, Mirowska KK, Mewada A (2012). Effect of long-term exposure to lower low-density lipoprotein cholesterol beginning early in life on the risk of coronary heart disease: a Mendelian randomization analysis. J Am Coll Cardiol.

[46] Voight BF, Peloso GM, Orho-Melander M, Frikke-Schmidt R, Barbalic M, Jensen MK (2012). Plasma HDL cholesterol and risk of myocardial infarction: a mendelian randomisation study. Lancet..

[47] Cholesterol Treatment Trialists C, Mihaylova B, Emberson J, Blackwell L, Keech A, Simes J (2012). The effects of lowering LDL cholesterol with statin therapy in people at low risk of vascular disease: meta-analysis of individual data from 27 randomised trials. Lancet..

[48] Pokharel Y, Gosch K, Nambi V, Chan PS, Kosiborod M, Oetgen WJ (2016). Practice-level variation in statin use among patients with diabetes: insights from the PINNACLE Registry. J Am Coll Cardiol.

[49] Natarajan P, Peloso GM, Zekavat SM, Montasser M, Ganna A, Chaffin M (2018). Deep-coverage whole genome sequences and blood lipids among 16,324 individuals. Nat Commun.

[50] Ripatti P, Ramo JT, Mars NJ, Fu Y, Lin J, Soderlund S (2020). Polygenic hyperlipidemias and coronary artery disease risk. Circ Genom Precis Med.

