# Supplementary material for: Development of genome-wide polygenic risk scores for lipid traits and clinical applications for dyslipidemia, subclinical atherosclerosis, and diabetes cardiovascular complications among East Asians
Source: Genome Med. 2021 Feb 19;13:29. doi: 10.1186/s13073-021-00831-z (PMC7893928; doi:10.1186/s13073-021-00831-z)
Supplement: Supplementary file 1 — Additional file 1: Supplementary methods: 1) Hong Kong Diabetes Register TRS Study Group Members; 2) Hong Kong Diabetes Biobank Study Group Members; and 3) Cohort descriptions. Figure S1. Principal component analysis (PCA). Figure S2. Pooled correlations of each candidate polygenic risk scores with measured lipid traits. Figure S3. Proportion of phenotypic variance in lipid traits explained by each candidate polygenic risk scores. Figure S4. Geometric means of measured lipid traits stratified by the quintile of polygenic risk score with the best performance. Figure S5. Geometric means of measured lipid traits at baseline and follow-up, and three-year changes in lipid traits stratified by quintile of polygenic risk scores in adolescents. [file 13073_2021_831_MOESM1_ESM.docx]

**SUPPLEMENTARY METHODS**

**Hong Kong Diabetes Register TRS Study Group Members**

Ronald C.W. Ma^1,2,3,4^, Juliana C.N. Chan^1,2,3^, Yu Huang^5^, Hui-yao Lan^1,3^, Si Lok^3^, Brian Tomlinson^1^, Stephen K.W. Tsui^5^, Weichuan Yu^6^, Kevin Y.L. Yip^7^, Ting-fung Chan^8^, Xiaodan Fan^9^, Wing-yee So^1,2^, Cheuk-chun Szeto^1^, Nelson L.S. Tang^3^, Andrea O.Y. Luk^1,2,3^, Xiaoyu Tian^5^, Guozhi Jiang^1,2^, Claudia H.T. Tam^1,2^, Heung Man Lee^1^, Cadmon K.P. Lim^1,2^, Katie K.H. Chan^2^, Fangying Xie^1^, Alex C.W. Ng^1^, Grace P.Y. Cheung^1^, Ming-wai Yeung^1^, Shi Mai^5^, Fei Xie^1^, Wei Jiang^6^, Sen Zhang^6^, Pu Yu^6^, Meng Weng^6^

^1^ Department of Medicine and Therapeutics, The Chinese University of Hong Kong, Hong Kong

^2^ Hong Kong Institute of Diabetes and Obesity, The Chinese University of Hong Kong, Hong Kong

^3^ Li Ka Shing Institute of Health Sciences, The Chinese University of Hong Kong, Hong Kong

^4^ Integrated Bioinformatics Laboratory for Cancer Biology and Metabolic Diseases, The Chinese University of Hong Kong, Hong Kong

^5^ School of Biomedical Sciences, The Chinese University of Hong Kong

^6^ Department of Electronic and Computer Engineering, The Hong Kong University of Science and Technology, Hong Kong

^7^ Department of Computer Science and Engineering, The Chinese University of Hong Kong, Hong Kong

^8^ School of Life Sciences, The Chinese University of Hong Kong, Hong Kong

^9^ Department of Statistics, The Chinese University of Hong Kong, Hong Kong

**Hong Kong Diabetes Biobank Study Group Members**

Ronald C.W. Ma ^1,2,3,4^, Juliana C.N. Chan ^1,2,3,4^, Risa Ozaki^1,2^, Andrea O.Y. Luk^1,2,3,4^, Wing-yee So^1,2^, Ka-fai Lee^5^, Shing-chung Siu^6^, Grace Hui^6^, Chiu-chi Tsang^7^, Kam-piu Lau^8^, Jenny Y.Y. Leung^9^, Man-wo Tsang^10^, Grace Kam^10^, Elaine Cheung^10^, Ip-tim Lau^11^, June K.Y. Li^12^, Vincent T.F. Yeung^13^, Jo Jo Kwan^13^, Samuel Fung^14^, Stanley Lo^15^, Emmy Lau^15^, Yuk-lun Cheng^16^, Stephen K.W. Tsui^17^, Yu Huang^17^, Huiyao Lan^1,3^, Weichuan Yu^18^, Brian Tomlinson^1^, Si Lok^19^, Ting-fung Chan^20^, Kevin Y.L. Yip^21^, Cheuk-chun Szeto^1,3^, Xiaodan Fan^22^, Nelson L.S. Tang^3, 23^, Xiaoyu Tian^17^, Claudia H.T. Tam^1,2,4^, Guozhi Jiang^1,2,4^, Shi Mai^17^, Baoqi Fan^1,2,4^, Fei Xie^1^, Sen Zhang^18^, Pu Yu^18^, Meng Wang^18^, Heung-man Lee^1^, Cadmon K.P. Lim ^1,2,4^, Fangying Xie^1^, Alex C.W. Ng^1^, Grace P.Y. Cheung^1^, Alice P.S. Kong^1,2^, Elaine Y.K. Chow^1,2^, Ming-wai Yeung^1^, Chun-chung Chow^1^, Kitty K.T. Cheung^1^, Rebecca Y.M. Wong^1^, Hon-cheong So^17^, Katie K.H. Chan^1,2^, Chin-san Law^11^, Anthea K.Y. Lock^11^, Ingrid K.Y. Tsang^11^, Susanna C.P. Chan^11^, Yin-wah Chan^11^, Cherry Chiu^2^, Chi-sang Hung^10^, Cheuk-wah Ho^10^, Ivy H.Y. Ng^10^, Maria W.H. Mak^7^, Kai-man Lee^7^, Candy H.S. Leung^13^, Ka-wah Lee^12^, Hui-ming Chan^12^, Winnie Wat^15^, Tracy Lau^15^, Cheuk-yiu Law^14^, Ryan H.Y. Chan^14^, Candice Lau^1^, Pearl Tsang^1^, Vince Chan^1^, Lap-ying Ho^1^, Eva Wong^1^, Josephine Chan^1^, Sau-fung Lam^1^, Jessy Pang^1^, Yee-mui Lee^1^

^1^ Department of Medicine and Therapeutics, The Chinese University of Hong Kong, Hong Kong

^2^ Hong Kong Institute of Diabetes and Obesity, The Chinese University of Hong Kong, Hong Kong

^3^ Li Ka Shing Institute of Health Sciences, The Chinese University of Hong Kong, Hong Kong

^4^ Chinese University of Hong Kong-Shanghai Jiao Tong University Joint Research Centre in Diabetes Genomics and Precision Medicine

^5^ Department of Medicine and Geriatrics, Kwong Wah Hospital, Hong Kong

^6^ Diabetes Centre, Tung Wah Eastern Hospital, Hong Kong

^7^ Diabetes and Education Centre, Alice Ho Miu Ling Nethersole Hospital, Hong Kong

^8^ North District Hospital, Hong Kong

^9^ Department of Medicine and Geriatrics, Ruttonjee Hospital, Hong Kong

^10^ Department of Medicine and Geriatrics, United Christian Hospital, Hong Kong

^11^ Tseung Kwan O Hospital, Hong Kong

^12^ Department of Medicine, Yan Chai Hospital, Hong Kong

^13^ Centre for Diabetes Education and Management, Our Lady of Maryknoll Hospital, Hong Kong

^14^ Department of Medicine and Geriatrics, Princess Margaret Hospital, Hong Kong

^15^ Department of Medicine, Pamela Youde Nethersole Eastern Hospital, Hong Kong

^16^ Department of Medicine, Alice Ho Miu Ling Nethersole Hospital, Hong Kong

^17^ School of Biomedical Sciences, The Chinese University of Hong Kong

^18^ Department of Electronic and Computer Engineering, Hong Kong University of Science and Technology, Hong Kong

^19^ The Centre for Applied Genomics, The Hospital for Sick Children, Toronto, Canada

^20^ School of Life Sciences, The Chinese University of Hong Kong, Hong Kong

^21^ Department of Computer Science and Engineering, The Chinese University of Hong Kong, Hong Kong

^22^ Department of Statistics, The Chinese University of Hong Kong, Hong Kong

^23^ Department of Chemical Pathology, The Chinese University of Hong Kong, Hong Kong

**Cohort descriptions**

*Cohorts of children and adult women*

Study methods of these cohorts have been described previously (1). The objective of the Hyperglycaemia and Adverse Pregnancy Outcome (HAPO) study was to clarify the risks of adverse pregnancy outcomes associated with maternal hyperglycemia below the level of overt diabetes mellitus by studying a heterogeneous, multinational, multicultural, ethnically diverse cohort of approximately 25,000 pregnant women with medical caregivers ‘blinded’ to the status of glucose tolerance (2). The HAPO study at the Hong Kong centre recruited 1,667 pregnant women with singleton pregnancy at the Prince of Wales Hospital from 2000 to 2006; all mothers underwent 75-g oral glucose tolerance test (OGTT) at 24^th^ -32^nd^ weeks of gestation. Women of non-Chinese ancestry, or women with glucose measurements beyond the setting of the HAPO study were excluded. Eligible mother-child pairs were invited to attend a follow-up assessment between 2009 and 2013 (approximately seven years postpartum). An additional 141 adult women who were diagnosed with gestational diabetes at our centre around the time of the HAPO study and received antenatal treatment were also invited to be part of the follow-up study (3).

*Cohort of adolescents*

This cohort originally consists of 2,309 adolescents recruited at baseline from a population-based school survey for risk factor assessment between February 2003 and February 2004 (4). To obtain a representative sample population of Hong Kong Chinese adolescents, we randomly selected 53 (out of 477) schools based on a full list of Chinese secondary schools in Hong Kong. From each participating school, six classes (Form one to Form six, equivalent to Grade seven to Grade 12 in the United States) were then randomly selected with one class from each form to obtain a proportional number of healthy participants, aged 12-20 years. Those with chronic illnesses such as diabetes, whether they were with or without drugs, were excluded from the study. All participants from the baseline study were invited to be part of the follow-up study conducted in 2006 (*n* = 692, response rate of 30.0%). Compared with adolescents who attended the follow-up visit, we found no evidence of discrepancy in baseline clinical characteristics between two groups, except HDL-C (Additional file 2: Table S17).

*Cohort of healthy adults and adults*

The cohort of 441 healthy adults and 426 adults were acquired from two sources. The first was a community-based health awareness and promotion program, the “Better Health for Better Hong Kong (BHBHK)” Campaign which commenced in 2000 and hospital staff (5). It specifically targets the low-income working population (i.e. income close to or below the median of the overall working population in Hong Kong) to raise their awareness regarding the importance of a healthy lifestyle using a wide range of education and health screening strategies. Between July 2000 and March 2002, participants were randomly selected by stratified random sampling in accordance to the distribution of occupational groups as recorded in the 1996 Hong Kong Population By-Census Report. The second source was a community-based pharmacogenetics study in hypertension and dyslipidaemia (6). DNA samples from 441 healthy adults and 426 adults were genotyped in two different phases using different genotyping platform.

*The Hong Kong Diabetes Register (HKDR) study*

The Hong Kong Diabetes Register (HKDR) baseline study ran from 1995 to 2007 and consists of >10,000 patients with diabetes (7). It was established as a quality improvement program and the study of outcomes for Chinese patients with diabetes at the Prince of Wales Hospital in Hong Kong, serving a population of over 1.2 million. These patients were referred from hospital-based specialty clinics, community clinics, and general practitioners. The Register consecutively enrolled patients with diabetes who were referred to the Diabetes Mellitus and Endocrine Centre for comprehensive assessment of diabetic complications and metabolic control. Type 2 Diabetes (T2D) was diagnosed according to the 1998 World Health Organization (WHO) criteria. Type 1 diabetic patients with acute ketotic presentation, or patients with non-Chinese or unknown nationality, or missing data on the type of diabetes, or continuous requirement of insulin within one year of diagnosis were excluded. In addition to detailed collection of clinical information and comprehensive assessment of diabetes complications at baseline according to the EURODIAB protocol, participants attended regular repeat diabetes complication assessment. Additional information on hospitalization, new diagnoses, prescription, and biochemical investigations are available and captured.

*The Hong Kong Diabetes Biobank (HKDB) phase 1 and 2 studies*

The Hong Kong Diabetes Biobank (HKDB) was established as a territory-wide diabetes registry and biobank for large-scale replication of genetic and epigenetic markers identified in the project since 2014 (<http://www.hongkongdiabetesbiobank.org/>, 31^st^ December, 2019). It recruited subjects through 11 participating diabetes centres and three renal units at major public hospitals across Hong Kong. Subjects with T2D undergoing routine diabetes complication screening assessment will be invited for recruitment into the Biobank. Similar methods and criteria were used for the recruitment as in HKDR. A total of 7,071 and 3,102 patients from HKDB were genotyped in phase 1 and phase 2 studies, respectively.

**References**

1. Tam WH, Ma RCW, Ozaki R, Li AM, Chan MHM, Yuen LY, et al. In Utero Exposure to Maternal Hyperglycemia Increases Childhood Cardiometabolic Risk in Offspring. Diabetes Care. 2017;40(5):679-86.

2. Group HSCR, Metzger BE, Lowe LP, Dyer AR, Trimble ER, Chaovarindr U, et al. Hyperglycemia and adverse pregnancy outcomes. N Engl J Med. 2008;358(19):1991-2002.

3. Tam WH, Ma RCW, Ozaki R, Li AM, Chan MHM, Yuen LY, et al. Antenatal treatment of gestational diabetes and offspring’s future cardiometabolic risk. the 9th International Symposium on Diabetes, Hypertension and Metabolic Syndrome and in Pregnancy; 8-12 March, 2017; Barcelona, Spain2017.

4. Ozaki R, Qiao Q, Wong GW, Chan MH, So WY, Tong PC, et al. Overweight, family history of diabetes and attending schools of lower academic grading are independent predictors for metabolic syndrome in Hong Kong Chinese adolescents. Arch Dis Child. 2007;92(3):224-8.

5. Ko GT, Chan JC, Chan AW, Wong PT, Hui SS, Tong SD, et al. Association between sleeping hours, working hours and obesity in Hong Kong Chinese: the 'better health for better Hong Kong' health promotion campaign. Int J Obes (Lond). 2007;31(2):254-60.

6. Hu M, Yang YL, Chan P, Tomlinson B. Pharmacogenetics of cutaneous flushing response to niacin/laropiprant combination in Hong Kong Chinese patients with dyslipidemia. Pharmacogenomics. 2015;16(12):1387-97.

7. Jiang G, Luk AOY, Tam CHT, Xie F, Carstensen B, Lau ESH, et al. Progression of diabetic kidney disease and trajectory of kidney function decline in Chinese patients with Type 2 diabetes. Kidney Int. 2019;95(1):178-87.

**Fig. S1**

**A) B) C)**

**D) E)**

**F) G) H)**

Principal component analysis (PCA) in each validation and testing cohorts. The PCA plots show the first two principal components, based on genotype data of 26 different populations from the 1000 Genomes Project, as well as each validation and testing cohorts in this study [A) children (*n* = 909); B) adolescents (*n* = 1,973); C) healthy adults (*n* = 441); D) adult women (*n* = 948); E) adults (*n* = 426); F) T2D patients in the HKDR study (*n* = 4,917); G) T2D patients in the HKDB phase 1 study (*n* = 1,941); and H) T2D patients in the HKDB phase 2 study (*n* = 865)]. The 26 populations from the 1000 Genomes Project have been divided into 5 super populations: 1) African (AFR) includes Yoruba in Ibadan, Nigeria, Luhya in Webuye, Kenya, Gambian in Western Divisions in the Gambia, Mende in Sierra Leone, Esan in Nigeria, Americans of African Ancestry in SW USA, and African Caribbeans in Barbados; 2) Ad Mixed American (AMR) includes Mexican Ancestry from Los Angeles USA, Puerto Ricans from Puerto Rico, Colombians from Medellin, and Colombia, Peruvians from Lima, Peru; 3) South Asian (SAS) includes Gujarati Indian from Houston, Texas, Punjabi from Lahore, Pakistan, Bengali from Bangladesh, Sri Lankan Tamil from the UK, and Indian Telugu from the UK; 4) European (EUR) includes Utah Residents (CEPH) with Northern and Western European Ancestry, Toscani in Italia, Finnish in Finland, British in England and Scotland, and Iberian Population in Spain; and 5) East Asian (EAS) includes Han Chinese in Beijing, China, Japanese in Tokyo, Japan, Southern Han Chinese, Chinese Dai in Xishuangbanna, China, and Kinh in Ho Chi Minh City, Vietnam.

**Fig. S2**

**Validation datasets:**

**T2D patients:**

Pooled correlations of each candidate polygenic risk scores with measured lipid traits [(A) total cholesterol, (B) triglycerides, (C) HDL cholesterol, and (D) LDL cholesterol] in combined analysis in validation datasets and combined analysis in T2D patients, related to Table 1. Each of the 34 polygenic risk scores was calculated using a total of 4,271 individuals from four cohorts of the Chinese population. By using the data on association statistics for four lipid traits from the Biobank Japan Project and the linkage disequilibrium reference panel from the 1000 Genomes East Asians, the first 27 scores (plot of the left-hand side) were derived based on the pruning and thresholding approach using a range of provided thresholds of linkage disequilibrium r2 (0.2, 0.4, and 0.6) and p values (1, 0.5, 0.1, 0.05, 0.01, 1×10^-3^, 1×10^-4^, 1×10^-5^, and 5×10^-8^). Seven additional scores (plot of the right-hand side) were constructed based on the LDPred computation algorithm using a range of tuning parameters ρ (1, 0.3, 0.1, 0.03, 0.01, 3×10^-3^, and 1×10^-3^), which are the proportion of causal variants. In each score, Pearson correlations with observed levels were computed in each individual cohort. The pooled correlations were calculated using the Fisher Z transformation approach.

**Fig. S3**

**Validation datasets:**

**T2D patients:**

Proportion of phenotypic variance in lipid traits (total cholesterol, triglycerides, HDL cholesterol, and LDL cholesterol) explained by each candidate polygenic risk scores in A) children (*n* = 909); B) adolescents (*n* = 1,973); C) healthy adults (*n* = 441); D) adult women (*n* = 948); E) T2D patients in the HKDR study (*n* = 4,917); F) T2D patients in the HKDB phase 1 study (*n* = 1,941); and G) T2D patients in the HKDB phase 2 study (*n* = 865), related to Table 1. Each of the 34 polygenic risk scores was calculated using a total of 4,271 individuals from four cohorts of the Chinese population. By using the data on association statistics for four lipid traits from the Biobank Japan Project and the linkage disequilibrium reference panel from the 1000 Genomes East Asians, the first 27 scores were derived based on the pruning and thresholding approach using a range of provided thresholds of linkage disequilibrium r2 (0.2, 0.4, and 0.6) and p values (1, 0.5, 0.1, 0.05, 0.01, 1×10^-3^, 1×10^-4^, 1×10^-5^, and 5×10^-8^). Seven additional scores were constructed based on the LDPred computation algorithm using a range of tuning parameters ρ (1, 0.3, 0.1, 0.03, 0.01, 3×10^-3^, and 1×10^-3^), which are the proportion of causal variants. The proportion of variance for a lipid trait explained by the corresponding candidate polygenic risk score was computed as the *R^2^* obtained from a full model including both PRS and covariates (PCs, sex, age, and BMI) minus the *R^2^* obtained from a model including covariates alone.

**Fig. S4**

**Validation datasets:**

**Testing datasets:**

Geometric means of measured lipid traits (total cholesterol, triglycerides, HDL cholesterol, and LDL cholesterol) stratified by the quintile of polygenic risk scores with the best performance (PRS_TC_, PRS_TG_, PRS_HDL_, and PRS_LDL_) in A) children (*n* = 909); B) adolescents (*n* = 1973); C) healthy adults (*n* = 441); D) adult women (*n* = 948); E) adults (*n* = 426); F) T2D patients in the HKDR study (*n* = 4917); G) T2D patients in the HKDB phase 1 study (*n* = 1941); and H) T2D patients in the HKDB phase 2 study (*n* = 865), related to Table 1. Within each cohort, *P_linear_* is the *p* value testing for a linear trend across five quintiles of polygenic risk score. *P_top_* is the *p* value testing for the association of a high polygenic risk score with corresponding lipid trait by comparing the top 20% of the distribution with the remaining 80% of the distribution. *P_bottom_* is the *p* value testing for the association of a low polygenic risk score with corresponding lipid trait by comparing the bottom 20% of the distribution with the remaining 80% of the distribution. All *p* values were obtained from linear regression with adjustments of principal components, sex, age, and body mass index. Lipid traits were natural log transformed for the linear regression analysis.

**Fig. S5**

Geometric means of measured lipid traits at baseline and follow-up, and three-year changes in lipid traits [(A) total cholesterol, (B) triglycerides, (C) HDL cholesterol, and (D) LDL cholesterol] stratified by quintile of polygenic risk scores in adolescents (*n* = 620), related to Table 2. Lipid traits at baseline and follow-up were natural log (*ln*) transformed. The three-year changes in lipid traits was transformed as *ln*(Y+1). *P_linear_* is the *p* value testing for a linear trend across five quintiles of polygenic risk score. *P_top_* is the *p* value testing for the association of a high polygenic risk score with corresponding lipid trait by comparing the top 20% of the distribution with the remaining 80% of the distribution. *P_bottom_* is the *p* value testing for the association of a low polygenic risk score with corresponding lipid trait by comparing the bottom 20% of the distribution with the remaining 80% of the distribution. Associations for quintile of polygenic risk scores with lipid traits at baseline and follow-up, and three-year changes in lipid traits were assessed by linear regression with the adjustments for models 1, 2, and 3, respectively. Model 1: principal components, sex, age at baseline, and BMI at baseline. Model 2: principal components, sex, age at follow-up, BMI at baseline and follow-up. Model 3: Model 2 + lipid trait at baseline.
